# Supplementary material for: A Systematic Targeted Genetic Screen Identifies Proteins Involved in Cytoadherence of the Malaria Parasite P. falciparum
Source: Mol Microbiol. 2025 Jan 20;123(4):330–43. doi: 10.1111/mmi.15337 (PMC11976115; doi:10.1111/mmi.15337)
Supplement: Supplementary file 1 — Data S1 Generation of glmS knockdown cell lines. (Top) Strategy to generate glmS knockdown cell lines via SLI. The native genomic locus was modified to add an additional C‐terminal skip‐peptide (S) and HA‐tag or GFP‐tag to the GOI using homologous recombination and selection‐linked integration (SLI). UTR, untranslated region. Primers used for integration PCRs are indicated by arrows. (Lower panels) Verification of M9‐ and glmS knockdown cell lines via integration‐PCR. GlmS and M9 cell lines were tested for 5′ (primer A and B) and 3′ integration (primer C and D), episomal plasmids (primer C and B) and wild‐type‐locus (primer A and D). CS2, WT‐positive control; ddH20, negative control. Additionally, the integrity of the subtelomeric regions of chromosome 2, which harbours kahsp40, emp3 and kahrp, was verified by PCR and restriction digest. S2. Overview of gene regions targeted and primer binding sites. S3. Primers and antibodies used in this study. S4. Sequence analysis. S5. Middle section of deconvoluted Z‐stack from Figure 1. S6. Deconvoluted Z‐stack movie from Figure 1. S7. Solubility analysis. Infected erythrocytes were lysed in water by repeated freeze‐thawing, separated into a supernatant (SN) and pellet (P) fraction by centrifugation and prepared for analysis by Western blot. A total of 1 × 10^7^ parasite equivalents were loaded per lane. Positive controls (left lanes) are total parasite extracts. Antibodies against PfAldolase were used as a control for the soluble fraction and PfEXP1 for membrane‐bound proteins. S8. Light microscopy of parasite‐infected erythrocytes. Cell morphology and cell cycle progression of glmS and M9 cell lines were monitored for 72 h by imaging Giemsa‐stained blood smears of parasites at the time points and with the GlcN concentrations indicated. Scale bar, 5 μm. All images are representative of at least 10 independent observations. S9. Localisation of other exported proteins in M9 cell lines. Immunofluorescent localisation of PfKAHRP, P [file MMI-123-330-s001.zip › S10_Binding_Data.pdf]

0300 glmS CSA binding assay

Experiment I						
	0mM			2.5mM		
CSA	142	174	180	170	100	83
CSASOL	50	68	29	21	70	57
normalised to the highest value of CSA 0 mM						
CSA	0,78888889	0,96666667	1	0,94444444	0,55555556	0,46111111
CSA SOL	0,27777778	0,37777778	0,16111111	0,11666667	0,38888889	0,31666667

Experiment II						
	0mM			2.5mM		
CSA	593	803	814	584	580	519
CSASOL	58	18	60	123	28	49
normalised to the highest value of CSA 0 mM						
CSA	0,72850123	0,98648649	1	0,71744472	0,71253071	0,63759214
CSA SOL	0,07125307	0,02211302	0,07371007	0,15110565	0,03439803	0,06019656

Experiment III						
	0mM			2.5mM		
CSA	3	904	1006	626	410	373
CSASOL	53	79	64	47	55	52
normalised to the highest value of CSA 0 mM						
CSA	0,00298211	0,89860835	1	0,6222664	0,40755467	0,37077535
CSA SOL	0,0526839	0,07852883	0,06361829	0,04671968	0,05467197	0,05168986

0600 glmS CSA binding assay

Experiment I						
	0mM			2.5mM		
CSA	263	295	361	124	363	158
CSASOL	89	97	88	51	46	55
normalised to the highest value of CSA 0 mM						
CSA	0,72853186	0,81717452	1	0,3434903	1,00554017	0,43767313
CSA SOL	0,2465374	0,26869806	0,24376731	0,14127424	0,12742382	0,15235457

Experiment II						
	0mM			2.5mM		
CSA	1010	939	902	307	1831	1063
CSASOL	171	9	44	28	7	24
normalised to the highest value of CSA 0 mM						
CSA	1	0,92970297	0,89306931	0,3039604	1,81287129	1,05247525
CSA SOL	0,16930693	0,00891089	0,04356436	0,02772277	0,00693069	0,02376238

Experiment III						
	0mM			2.5mM		
CSA	1379	1691	2029	1075	348	360
CSASOL	101	246	92	43	69	41
normalised to the highest value of CSA 0 mM						
CSA	0,67964515	0,83341548	1	0,52981764	0,17151306	0,1774273
CSA SOL	0,04977822	0,12124199	0,04534253	0,02119271	0,0340069	0,020207

1600 glmS CSA binding assay

Experiment I						
	0mM			2.5mM		
CSA	344	270	346	131	48	107
CSASOL	62	49	21	28	25	59
normalised to the highest value of CSA 0 mM						
CSA	0,99421965	0,78034682	1	0,37861272	0,13872832	0,30924855
CSA SOL	0,17919075	0,1416185	0,06069364	0,08092486	0,07225434	0,17052023

Experiment II						
	0mM			2.5mM		
CSA	2241	2151	2624	1200	1358	847
CSASOL	160	338	357	252	183	100
normalised to the highest value of CSA 0 mM						
CSA	0,85403963	0,81974085	1	0,45731707	0,51753049	0,32278963
CSA SOL	0,06097561	0,12881098	0,13605183	0,09603659	0,06974085	0,03810976

Experiment III						
	0mM			2.5mM		
CSA	509	328	343	273	279	440
CSASOL	20	32	29	115	40	43
CSA	1	0,64440079	0,67387033	0,53634578	0,5481336	0,86444008
CSA SOL	0,03929273	0,06286837	0,05697446	0,2259332	0,07858546	0,08447937

3300 glmS CSA binding assay

Experiment I						
	0mM			2.5mM		
CSA	1549	1474	926	1439	1328	991
CSASOL	154	268	164	438	386	553
normalised to the highest value of CSA 0 mM						
CSA	1	0,95158167	0,59780504	0,92898644	0,85732731	0,63976759
CSA SOL	0,09941898	0,17301485	0,10587476	0,28276307	0,24919303	0,35700452

Experiment II						
	0mM			2.5mM		
CSA	229	202	194	193	253	136
CSASOL	11	35	68	40	11	84
normalised to the highest value of CSA 0 mM						
CSA	1	0,88209607	0,84716157	0,84279476	1,10480349	0,59388646
CSA SOL	0,04803493	0,15283843	0,29694323	0,17467249	0,04803493	0,36681223

Experiment III						
	0mM			2.5mM		
CSA	276	371	353	173	216	161
CSASOL	49	67	53	47	38	47
normalised to the highest value of CSA 0 mM						
CSA	0,74393531	1	0,95148248	0,46630728	0,58221024	0,43396226
CSA SOL	0,13207547	0,18059299	0,14285714	0,12668464	0,10242588	0,12668464
