# Supplementary material for: A Systematic Targeted Genetic Screen Identifies Proteins Involved in Cytoadherence of the Malaria Parasite P. falciparum
Source: Mol Microbiol. 2025 Jan 20;123(4):330–43. doi: 10.1111/mmi.15337 (PMC11976115; doi:10.1111/mmi.15337)
Supplement: Supplementary file 1 — Data S1 Generation of glmS knockdown cell lines. (Top) Strategy to generate glmS knockdown cell lines via SLI. The native genomic locus was modified to add an additional C‐terminal skip‐peptide (S) and HA‐tag or GFP‐tag to the GOI using homologous recombination and selection‐linked integration (SLI). UTR, untranslated region. Primers used for integration PCRs are indicated by arrows. (Lower panels) Verification of M9‐ and glmS knockdown cell lines via integration‐PCR. GlmS and M9 cell lines were tested for 5′ (primer A and B) and 3′ integration (primer C and D), episomal plasmids (primer C and B) and wild‐type‐locus (primer A and D). CS2, WT‐positive control; ddH20, negative control. Additionally, the integrity of the subtelomeric regions of chromosome 2, which harbours kahsp40, emp3 and kahrp, was verified by PCR and restriction digest. S2. Overview of gene regions targeted and primer binding sites. S3. Primers and antibodies used in this study. S4. Sequence analysis. S5. Middle section of deconvoluted Z‐stack from Figure 1. S6. Deconvoluted Z‐stack movie from Figure 1. S7. Solubility analysis. Infected erythrocytes were lysed in water by repeated freeze‐thawing, separated into a supernatant (SN) and pellet (P) fraction by centrifugation and prepared for analysis by Western blot. A total of 1 × 10^7^ parasite equivalents were loaded per lane. Positive controls (left lanes) are total parasite extracts. Antibodies against PfAldolase were used as a control for the soluble fraction and PfEXP1 for membrane‐bound proteins. S8. Light microscopy of parasite‐infected erythrocytes. Cell morphology and cell cycle progression of glmS and M9 cell lines were monitored for 72 h by imaging Giemsa‐stained blood smears of parasites at the time points and with the GlcN concentrations indicated. Scale bar, 5 μm. All images are representative of at least 10 independent observations. S9. Localisation of other exported proteins in M9 cell lines. Immunofluorescent localisation of PfKAHRP, P [file MMI-123-330-s001.zip › S2_Targeted gene regions.pdf]

S2 Targeted Gene Regions

Targeting regions highlighted in grey. Lower case are introns.Red text shows primer binding regions.

PF3D7_0113300_gDNA Targeted region 981 bp

ATGAAAAATATCAAAAATATGAAAAATATAAAAAGTGAAGGTTTCATTTTTTTTGTACTTGTTTTTTTTATATGT
ATTATTTTTGGTTGTATATATGAAAGTCTGCACGAGgtaataataaaacataaaaatataaagacaaaaatgaag
taatagcatataagtgtatatatatatatatatatatatatatatatatatatatttatatttatatttatatttat
ttatttatttatatttattttctttacttattacatttctttttatttattttttttcctttttgtagGGTCCAT
ATAAAAAAACCCTTAACAGTCTTCATGAATCAACAAAGTATAGACATTTTAATAAGATCAGGTTATTAACAGAAT
ATAAAGACACATTACAAATTAAAGTAGAACAAAAATCTTTACGTGATTATGTAAATAATGATCGTTATAATAATG
TAAATACGAATGATTATACTTCATACAAAGATAAGGGAGAGCAATTTAATGATACCATTTGTGTTGTTGATAAAA
AAAAAGAGAATGTAACTATTAATAATGAAGAAGAATGTAATAAAAATTTTTATCAGTATTTACAATATCTTGAAC
ATAACAACAAACAGGACAATAAATATGAAGAAACGAATTATTTCTTACAGGGAAATGATAAACATATAGATAGTG
AACATAATGGAATAAATAAAATGTACAAAGAAACAATACATAAAACACTAACATCTGATGTATCAACAGAAAATT
CATATACTCATAATAATTCAAGAGATGATGAACCTCAAAATGGAAAACGCACATATAATAATCAATCTAATAATA
ATTTGCCATATGATAATTCTTCTTATAATATATCACCTTATCATGGTCCAAATAATAATGTACCTTATAATAAAT
CAAACAATTTTGAACAATGTAACACTCAAGATAATAAACACTGTAATGATCTAGACACATATCATACGTGCTATG
GACCTGATAATTATCCACAAAAATATGATAATTATCGACAAGAATGTGATAATTATCGACAAGAATGTGATAATT
ATCGACAAGAATATGATAATTATCCACAAAAATATGATAATTATCGACAAGAATGTGATAATTATCGACAAGAAT
ATGATAATTATCCACATGGGTTTGATAACTATCCACGTGGGTTTGATAACTATCCACATGGATATGATAATCATC
CCCATAGACCGCATATTTATCCACATGGGTTTGATAATCATCCCCATAGACCTCATATGTATCCACATAATTTTC
CGATGAGAAATGAATCAGTGGGGGGCCCTTATTACAGACCCCCTCATATAATAGAACGATCGAATTATTATAAAA
ATCCAAAAAAGGCCCCTCACAATATGATGTTACCATGTGATACCATGAAAGATAATAAATCGATATGTGATGAAC
AAAATTTTCAACGAGAATTAGAAAAAATAATAAAAAAAAAATAATTTACAAAATGGTAATATCAGAGATAATCATG
ACACAAGAATTAATGATTATAATAAAAGATTGACAGAATATAATAAAAGATTAACAGAATATAATAAAAGATTAA
CTGAATATACCAAAAGATTAAACGAACATTATAAAAGAAATGGTTATAATATACAAAATAGACAAAATAGTATAG
AACGTGCACAATCAAATGATGTAGTTTTATATGGACATAATTTTCAAAATGCTTTTAGATATAAACAAAATACAC
GTTCGTATTATCCACATGTTAATTCAAACGAAGCAACACATCATCAAAAAACAATGTATTTTACTCAACAAAATA
ATTATTCAAGAGAAGAATATCCAATAAAAAGTGAACAACATTTGTATCATGTAAAATCAAAACGTTTGGAAAAAA
AATTGTATGATTATCAAAATGGTACAAACCCTGTAACCAACTTTTTAGAGCGCCATTTTTAA

PF3D7_0220300_gDNA Targeted region 778 bp

ATGAATATGTTTTTTTTATTCATAAAAATTTTTATTTTTTCCATTTTCACTATAAACCTTAAATTAACTAATAGA
gtaacccacaaaaaaaaaaaaaaaaaaaaaaaaaaaaaaccatatatataaatataaatatatatatatatata
tatatatatatatatatatataataaaatgaagtaatatatttattataatgcttcattaattattcttacatat
atttttattcccccccttttcgtttttcttttatagAATGATTATAATATACCCTACAAAGGAAAAAAGAATC
TCCTAGGAAAAAGATTAGGTTTAATATCATGTAGAACCTTAACAGAAGTTTATGATGCCTTTAACGATGCAACAG
TAAAAATGCTAGATATTAGTTTTGATTCTTCGAAAGCGAGTACCACAAAAACTCGAAGATTTATTCATGACAGAT
ATTCAACAGCACCATATGAATTAATAAAGCGTAAACCCAAAAAAAAAAAAATCTCCATTTTCTAAAATGAAGGTGA
AATTTGTAAGATATTTTCAAAAAATTAATTATATATTAAATGAAATATTATATTTTTTTATGGATAACACAAACT
GCTTTTTTTTACCAACGAAATATATATTAGCACCATTTGTTTTCACATTCAAAATTCTTTATGACATTATAGCTT
TTATAGCAATTGTTTTTATCTTGGTAATCATAATTATTTATACACTCATAAAAAAAAATGTGTCTAAGAATGCATT
ATAGTGATAGTATGCAAAATTTAAGATCAAGGAAGAATCATGAAGCCAAGGTTAAAATATACAAACCCGAAGCAT
GA

PF3D7_0220600_gDNA Targeted region 758 bp

ATGGGTTATTCAAATAATAAATTTAATATTTTTACATTATGGAATAATATTATTTTGTATTTCATTCTTATAGTT
ACATTTACCTTTTATAACAAGgtatgaaaagtaaaaaaaattgaatatatttgtttatatatatatatatata
tatataattttatttaaagttttatttaatatattatattatatatatatatatatatatttttttttttttttt
ttttttatagGATTTGTTTAATAAGTATAATGGTGAAAAATCAAACATAGGGGCTTCATTTAATTTTGGAAATAA
TAGATCATTAGCAGAATATTATAACAACAAAGATGGGTACAATGTATTAAGAGTAAATTTGGATCACAAAAATCT
TAAAGATGTCTTAGGGAATATGCATCCTGAAATAAAAATGGTAGAAGTTGATTCAGAAAGTGTATGCCCAGGTAC
AAATGAAGTTAATTTAAAGGTTGTTACGAATATACCACCAGATATGATTAAAGTAAATGCAACAAGTGAAAATAT
GTCAGTAGGACAATGGGATTATATTATGCAATATTATGGTCAATCAACACCTAAGGAAGTATCTAAATTAGATTC
GGAAGTAAAAGACAAAATAGAAAAGAAAATTAAAAAGAAGAAAAGAAAAACACCTTTAATAAGATATATTGCAGA
ACTTGTAGGTTATGGTATTATTTTCATTCCTGGTTTTCCTGTTCTTGTTGGTATAGTAAGTGTTGGATTCTGTAT
ATTGATCTTTATGGGTAAAAAATCTGCAAAAAATTATTTTAGTACAATTAAGAAATGGCTTTTTTAA

PF3D7_0301600_gDNA Targeted region 1128 bp

ATGATAACTAAAAATTATAATATATCAAAAAATGTTATAAGACAGATAGGAATAGGAGTGAATAAATCTATTTGT
TTTACATGTAAAAATATAAATATATTGGGAAAATGTCAGAAAATATGTATAAAACATTTTTTGATATTTAATAAA
ATTCTGTTGTTATTTATTTTAGTATGGATATTTCAATATAGTAATGAAGAAgtaagataataataaactgattat
tttaatatatatatatatatatatatatatatatatatatatatatatatatgtgtatatttatttatctattta
ttttttttgcgtttattattttttaattttcgtttttttttctttattatagTTTACCTGTAAAGGTAGTAGATCA
ACAAAGCAAGATGAAGAGAAAGCATCCTTAAGATTTTATAGATTATTAGCCCAAAGTTATGTATTTAATTCCCGT
TTGGATGGATTTCATAATTATTCTAATGCAGAGAATGAAAGGATGTTTATGCGAAATTATATGAATGATGGTTTG
TTTAAATCCCAAGCGTATAATGAATCAGAAAAAAGAGATAATTTAAGAAATAATATATACAATGAACAAAATGAT
TTTGCTAAAGAAGGATATTCGAGAGAATATGACAGCAAATATAACTCTGCAGAATTTGGTACATCTAGAAAGAAA
AATGATGGAAGAAATCCATTTAATTATCAATCGAATAATGAAAATAATAAACCTGCTTCATATATGAATGAGGAT
ATTTATAATAAAAATTATTATAACCAAAGAGAAAATATAGATGAATTAAATAAAAAATTACATGATGATTGGTCG
AATTTTTTAAATGAAGATTATTCTAATGAATTTGAAAGTAATAATTTTAATTCACCACATTATGATACATATAAA
TCAGAATATGATACGTACAAATCAGAATATGATACATATAAATCACAATATGATACATATAAATCACAATATGAT
GATAGAAATAAATATTCCTATCCATCAAGAGAAGGGAATTATGTACCTCCTTTTATGGATGAAAATGATAATAGA
TTAAATAACAATTATAGAAGTGGATTTGATGACACTAAAAAGAATAATATAAATGATGATAAAGGAATGAATTTT
GTAAGAGATGAATTTTACGATGAATATAATAATAATAATATGAATACATCACCGGAATATATGTTCTCACAAGAA
TGGGATAATCAAAATACACATGATGGATTTCCATCAGGATGGAATAATTACAATAGTGAAAATAGTGATCATAAA
GATCAAATAAAGGTTGAAACGCCATATATTAGGGTAGTTGAAGAAGTAAATGATGAATCAAATAATAAAAGAGAT
AATTATGATACGTTAAATAAAGATTCATCATGTAATAGAAATGAAAAAATGGAATACTATTTCCAATCAGATAAA
TTTCCAGTTGATTGTAAAGGCATATATACGAATTCTAATATAATAGTAGAAGAATATGATACTCCTGTTGATTTA
AAAGAAACCAATGATAATTTTTATTCATTTAATGAAAAATTAAATAATGAGAGACAAAAAAAATATATGCGAGAA
GATAAAGAATTTATAAATGAAATGGGACATATGTCATCAACATCGTCAAATTCTCCGTCATCACATTTTCCGTCA
CCACACTCACAACCCTCGTTTATACCAGAAACGTTTCAAGATTCCAATATTGAATCACCAGAATTTGAATTACCG
ACTTTTGAATCAGCAACTGTTCATACATCAGAATATGAACTACCGAATTTTGATATATTAGAATATGATTTACCA
CATTTTGATCCATCAGAATTTAAACCTGAAATTTTTGATGAATCAGAATTTAAACCTGAAATTTTTGATGAATCA
GAATTTAAACCATCAGTTTTTGATCCGTCCGAATATAAAAATTCACCATTTAAAGAATCAAAAATTTCAACATCA
TCACATTCAACACGATCTTATGAAAATGAATTTAACTTAAGTACTGATAGAAGAAATAGTGAATCGTCTTCTAGA
ATACACGTTGATGATGAAAATTATAAACGTTTGAAAAAAATATTAAGTAACTTATGTTCAAGTAGGGAAAAAATA
TATGATCTTAGTAAAGAAGAGAAAGAATTCCTCGTAAAAATGCTACAATTAGATTATGAAGATTTTAATTTTAAT
TATAGAGGAAACACACGTAGTTATAATGATTCATCACCATTCACAAATTTTGAATTAAAACGAATACATTTAAAA
ATAAAAGCATTTATATACAAATGTCTTTTACGTTTATTACAATCTTATGAAAAATCAACTGAAAGTCGTGTAGGC
AGTAATATATATATGATATATATTAG
