# Supplementary material for: A Systematic Targeted Genetic Screen Identifies Proteins Involved in Cytoadherence of the Malaria Parasite P. falciparum
Source: Mol Microbiol. 2025 Jan 20;123(4):330–43. doi: 10.1111/mmi.15337 (PMC11976115; doi:10.1111/mmi.15337)
Supplement: Supplementary file 1 — Data S1 Generation of glmS knockdown cell lines. (Top) Strategy to generate glmS knockdown cell lines via SLI. The native genomic locus was modified to add an additional C‐terminal skip‐peptide (S) and HA‐tag or GFP‐tag to the GOI using homologous recombination and selection‐linked integration (SLI). UTR, untranslated region. Primers used for integration PCRs are indicated by arrows. (Lower panels) Verification of M9‐ and glmS knockdown cell lines via integration‐PCR. GlmS and M9 cell lines were tested for 5′ (primer A and B) and 3′ integration (primer C and D), episomal plasmids (primer C and B) and wild‐type‐locus (primer A and D). CS2, WT‐positive control; ddH20, negative control. Additionally, the integrity of the subtelomeric regions of chromosome 2, which harbours kahsp40, emp3 and kahrp, was verified by PCR and restriction digest. S2. Overview of gene regions targeted and primer binding sites. S3. Primers and antibodies used in this study. S4. Sequence analysis. S5. Middle section of deconvoluted Z‐stack from Figure 1. S6. Deconvoluted Z‐stack movie from Figure 1. S7. Solubility analysis. Infected erythrocytes were lysed in water by repeated freeze‐thawing, separated into a supernatant (SN) and pellet (P) fraction by centrifugation and prepared for analysis by Western blot. A total of 1 × 10^7^ parasite equivalents were loaded per lane. Positive controls (left lanes) are total parasite extracts. Antibodies against PfAldolase were used as a control for the soluble fraction and PfEXP1 for membrane‐bound proteins. S8. Light microscopy of parasite‐infected erythrocytes. Cell morphology and cell cycle progression of glmS and M9 cell lines were monitored for 72 h by imaging Giemsa‐stained blood smears of parasites at the time points and with the GlcN concentrations indicated. Scale bar, 5 μm. All images are representative of at least 10 independent observations. S9. Localisation of other exported proteins in M9 cell lines. Immunofluorescent localisation of PfKAHRP, P [file MMI-123-330-s001.zip › S4_Sequence_Analysis.pdf]

Predicted protein sequences POI
Predicted TM domain in blue
PEXEL sequence in red

>300
MNMFFLFIKIFIFSIFTINLKLTNRNDYNIPYKGKKNLLGKRLGLISCRTLTEVYDAFNDATVKMLDISFDSSKASTTKT 25kDa pI 10.58
RRFIHDRYSTAPYELIKRKPKKKKSPFSKMKVKFVRYFQKINYILNEILYFFMDNTNCFFLPTKYILAPFVFTFKILYDI
IAFIAIVFILVIIIIYTLIKKMCLRMHYSDSMQNLRSRKNHEAKVKIYKPEA

>600
MGYSNNKFNIFTLWNNIILYFILIVTFTFYNKDLFNKYNGEKSNIGASFNFGNNRSLAEYYNNKDGYNVLRVNLDHKNLK 26kDa pI 9.83
DVLGNMHPEIKMVEVDSESVCPGTNEVNLKVVTNIPPDMIKVNATSENMSVGQWDYIMQYYGQSTPKEVSKLDSEVKDKI
EKKIKKKKRKTPLIRYIAELVGYGIIFIPGFPVLVGIVSVGFCILIFMGKKSAKNYFSTIKKWLF

>1600
MITKNYNISKNVIRQIGIGVNKSICFTCKNINILGKCQKICIKHFLIFNKILLLFILVWIFQYSNEEFTCKGSRSTKQDE 88kDa pI 4.77
EKASLRFYRLLAQSYVFNSRLDGFHNYSNAENERMFMRNYMNDGLFKSQAYNESEKRDNLRNNIYNEQNDFAKEGYSREY
DSKYNSAEFGTSRKKNDGRNPFNYQSNNENNKPASYMNEDIYNKNYYNQRENIDELNKKLHDDWSNFLNEDYSNEFESNN
FNSPHYDTYKSEYDTYKSEYDTYKSQYDTYKSQYDDRNKYSYPSREGNYVPPFMDENDNRLNNNYRSGFDDTKKNNINDD
KGMNFVRDEFYDEYNNNNMNTSPEYMFSQEWDNQNTHDGFPSGWNNYNSENSDHKDQIKVETPYIRVVEEVNDESNNKRD
NYDTLNKDSSCNRNEKMEYYFQSDKFPVDCKGIYTNSNIIVEEYDTPVDLKETNDNFYSFNEKLNNERQKKYMREDKEFI
NEMGHMSSTSSNSPSSHFPSPHSQPSFIPETFQDSNIESPEFELPTFESATVHTSEYELPNFDILEYDLPHFDPSEFKPE
IFDESEFKPEIFDESEFKPSVFDPSEYKNSPFKESKISTSSHSTRSYENEFNLSTDRRNSESSSRIHVDDENYKRLKKIL
SNLCSSREKIYDLSKEEKEFLVKMLQLDYEDFNFNYRGNTRSYNDSSPFTNFELKRIHLKIKAFIYKCLLRLLQSYEKST
ESRVGSNIYDIY

>3300
MKNIKNMKNIKSEGFIFFVLVFFICIIFGCIYESLHEGPYKKTLNSLHESTKYRHFNKIRLLTEYKDTLQIKVEQKSLRD 71kDa pI 7.98
YVNNDRYNNVNTNDYTSYKDKGEQFNDTICVVDKKKENVTINNEEECNKNFYQYLQYLEHNNKQDNKYEETNYFLQGNDK
HIDSEHNGINKMYKETIHKTLTSDVSTENSYTHNNSRDDEPQNGKRTYNNQSNNNLPYDNSSYNISPYHGPNNNVPYNKS
NNFEQCNTQDNKHCNDLDTYHTCYGPDNYPQKYDNYRQECDNYRQECDNYRQEYDNYPQKYDNYRQECDNYRQEYDNYPH
GFDNYPRGFDNYPHGYDNHPHRPHIYPHGFDNHPHRPHMYPHNFPMRNESVGGPYYRPPHIIERSNYYKNPKKAPHNMML
PCDTMKDNKSICDEQNFQRELEKIIKKNNLQNGNIRDNHDTRINDYNKRLTEYNKRLTEYNKRLTEYTKRLNEHYKRNGY
NIQNRQNSIERAQSNDVVLYGHNFQNAFRYKQNTRSYYPHVNSNEATHHQKTMYFTQQNNYSREEYPIKSEQHLYHVKSK
RLEKKLYDYQNGTNPVTNFLERHF

Alignment Syntenic Orthologues PF3D7_0301600

```
PADL01_0034200-t36_1-p1       MINKNYNISKNGIRQIGIRANMTICFTSKHINIIGKCQKKYIKHFLIFNKVLLLFVLVCI    60
PGABG01_0300800-t36_1-p1      MINKNYNISKNGIRQIGIRANRTICFTSKHINIIGKCQKKYIKHFLIFNKFLLLFILVSI    60
PGSY75_0301600-t31_1-p1       MINKNYNISKNGIRQIGIRANRTICFTSKHINIIGKCQKKYIKHFLIFNKVLLLFILVCI    60
PBLACG01_0301900-t36_1-p1     MITQNYNISKNVIRQIGIRANRSICFTCKHINILQKCQKIYIKHFLIFNKILLLFILVWI    60
PRG01_0304500-t36_1-p1        MVTKNYNISKNVIRQIGIGVNKSICFTFKNINILGKCQKICIKHFLIFNKILLLFILVWI    60
PRCDC_0300700.1-p1            MVTKNYNISKNVIRQIGIGVNKSICFTFKNINILGKCQKICIKHFLIFNKILLLFILVWI    60
PfML01_030006900-t41_1-p1     MITKNYNISKNVIRQIGIGVNKSICFTCKNINILGKCQKICIKHFLIFNKILLLFILVWI    60
PfGB4_030007500-t41_1-p1      MITKNYNISKNVIRQIGIGVNKSICFTCKNINILGKCQKICIKHFLIFNKILLLFILVWI    60
PfSD01_030006800-t41_1-p1     MITKNYNISKNVIRQIGIGVNKSICFTCKNINILGKCQKICIKHFLIFNKILLLFILVWI    60
PfKH02_030007200-t41_1-p1     MITKNYNISKNVIRQIGIGVNKSICFTCKNINILGKCQKICIKHFLIFNKILLLFILVWI    60
PfIT_030006400-t41_1-p1       MITKNYNISKNVIRQIGIGVNKSICFTCKNINILGKCQKICIKHFLIFNKILLLFILVWI    60
PfKH01_030006400-t41_1-p1     MITKNYNISKNVIRQIGIGVNKSICFTCKNINILGKCQKICIKHFLIFNKILLLFILVWI    60
PfNF135_030006800.1-p1        MITKNYNISKNVIRQIGIGVNKSICFTCKNINILGKCQKICIKHFLIFNKILLLFILVWI    60
PfCD01_030007000-t41_1-p1     MITKNYNISKNVIRQIGIGVNKSICFTCKNINILGKCQKICIKHFLIFNKILLLFILVWI    60
PfGN01_030007300-t41_1-p1     MITKNYNISKNVIRQIGIGVNKSICFTCKNINILGKCQKICIKHFLIFNKILLLFILVWI    60
PfKE01_030006300-t41_1-p1     MITKNYNISKNVIRQIGIGVNKSICFTCKNINILGKCQKICIKHFLIFNKILLLFILVWI    60
PfSN01_030007100-t41_1-p1     MITKNYNISKNVIRQIGIGVNKSICFTCKNINILGKCQKICIKHFLIFNKILLLFILVWI    60
PfTG01_030008300-t41_1-p1     MITKNYNISKNVIRQIGIGVNKSICFTCKNINILGKCQKICIKHFLIFNKILLLFILVWI    60
Pf7G8-2_000071200.1-p1        MITKNYNISKNVIRQIGIGVNKSICFTCKNINILGKCQKICIKHFLIFNKILLLFILVWI    60
Pf7G8_030007400-t41_1-p1      MITKNYNISKNVIRQIGIGVNKSICFTCKNINILGKCQKICIKHFLIFNKILLLFILVWI    60
PfHB3_030005500-t41_1-p1      MITKNYNISKNVIRQIGIGVNKSICFTCKNINILGKCQKICIKHFLIFNKILLLFILVWI    60
PF3D7_0301600.1-p1            MITKNYNISKNVIRQIGIGVNKSICFTCKNINILGKCQKICIKHFLIFNKILLLFILVWI    60
PfNF54_030006800.1-p1         MITKNYNISKNVIRQIGIGVNKSICFTCKNINILGKCQKICIKHFLIFNKILLLFILVWI    60
PfGA01_030008000-t41_1-p1     MITKNYNISKNVIRQIGIGVNKSICFTCKNINILGKCQKICIKHFLIFNKILLLFILVWI    60
PfDd2_030006600-t41_1-p1      MITKNYNISKNVIRQIGIGVNKSICFTCKNINILGKCQKICIKHFLIFNKILLLFILVWI    60
                              *:.:******* ****** .* :**** *:***: ****  *********.****:** *

PADL01_0034200-t36_1-p1       CQYSIDEITFNGSRSIKQDEEKASLRFNRLLAQSFVFNSRLDGSYNYSNVENERMYMPHY    120
PGABG01_0300800-t36_1-p1      CQYSIDEITFNGSRSTKQDEEKASLRFNRLLAQSFVFNSRLDGSYNYPNVENERMYMPHY    120
PGSY75_0301600-t31_1-p1       YQYSIDEITFNGSRSTKQDEEKASLRFNRLLAQSFVFNSRLDGSYNYPNVENERMYMPHY    120
PBLACG01_0301900-t36_1-p1     FQYSNEDFNCKGSRSTKHDEERASLRFYRLLAQSYIFNSRLDGSYNYSNVENERMYMRDY    120
PRG01_0304500-t36_1-p1        FQYSNEEFTCKGSRSTKQDEEKASLRFYRLLAQSYLFNSRLDGSYNYSNAENERMFMRNY    120
PRCDC_0300700.1-p1            FQYSNEEFTCKGSRSTKQDEEKASLRFYRLLAQSYLFNSRLDGSYNYSNAENERMFMRNY    120
PfML01_030006900-t41_1-p1     FQYSNEEFTCKGSRSTKQDEEKASLRFYRLLAQSYVFNSRLDGFHNYSNAENERMFMRNY    120
PfGB4_030007500-t41_1-p1      FQYSNEEFTCKGSRSTKQDEEKASLRFYRLLAQSYVFNSRLDGFHNYSNAENERMFMRNY    120
PfSD01_030006800-t41_1-p1     FQYSNEEFTCKGSRSTKQDEEKASLRFYRLLAQSYVFNSRLDGFHNYSNAENERMFMRNY    120
PfKH02_030007200-t41_1-p1     FQYSNEEFTCKGSRSTKQDEEKASLRFYRLLAQSYVFNSRLDGSHNYSNAENERMFMRNY    120
PfIT_030006400-t41_1-p1       FQYSNEEFTCKGSRSTKQDEEKASLRFYRLLAQSYVFNSRLDGSHNYSNAENERMFMRNY    120
PfKH01_030006400-t41_1-p1     FQYSNEEFTCKGSRSTKQDEEKASLRFYRLLAQSYVFNSRLDGSHNYSNAENERMFMRNY    120
PfNF135_030006800.1-p1        FQYSNEEFTCKGSRSTKQDEEKASLRFYRLLAQSYVFNSRLDGFHNYSNAENERMFMRNY    120
PfCD01_030007000-t41_1-p1     FQYSNEEFTCKGSRSTKQDEEKASLRFYRLLAQSYVFNSRLDGFHNYSNAENERMFMRNY    120
PfGN01_030007300-t41_1-p1     FQYSNEEFTCKGSRSTKQDEEKASLRFYRLLAQSYVFNSRLDGFHNYSNAENERMFMRNY    120
PfKE01_030006300-t41_1-p1     FQYSNEEFTCKGSRSTKQDEEKASLRFYRLLAQSYVFNSRLDGFHNYSNAENERMFMRNY    120
PfSN01_030007100-t41_1-p1     FQYSNEEFTCKGSRSTKQDEEKASLRFYRLLAQSYVFNSRLDGFHNYSNAENERMFMRNY    120
PfTG01_030008300-t41_1-p1     FQYSNEEFTCKGSRSTKQDEEKASLRFYRLLAQSYVFNSRLDGFHNYSNAENERMFMRNY    120
Pf7G8-2_000071200.1-p1        FQYSNEEFTCKGSRSTKQDEEKASLRFYRLLAQSYVFNSRLDGFHNYSNAENERMFMRNY    120
Pf7G8_030007400-t41_1-p1      FQYSNEEFTCKGSRSTKQDEEKASLRFYRLLAQSYVFNSRLDGFHNYSNAENERMFMRNY    120
PfHB3_030005500-t41_1-p1      FQYSNEEFTCKGSRSTKQDEEKASLRFYRLLAQSYVFNSRLDGFHNYSNAENERMFMRNY    120
PF3D7_0301600.1-p1            FQYSNEEFTCKGSRSTKQDEEKASLRFYRLLAQSYVFNSRLDGFHNYSNAENERMFMRNY    120
PfNF54_030006800.1-p1         FQYSNEEFTCKGSRSTKQDEEKASLRFYRLLAQSYVFNSRLDGFHNYSNAENERMFMRNY    120
PfGA01_030008000-t41_1-p1     FQYSNEEFTCKGSRSTKQDEEKASLRFYRLLAQSYVFNSRLDGFHNYSNAENERMFMRNY    120
PfDd2_030006600-t41_1-p1      FQYSNEEFTCKGSRSTKQDEEKASLRFYRLLAQSYVFNSRLDGSHNYSNAENERMFMRNY    120
                               *** :::. :**** *:***:***** ******::******* :** *.*****:* .*
```

```
PADL01_0034200-t36_1-p1        MNNNFFKSQAYNESEKRDNLRSKIYNEQNDFAKEGYSREYDSKYNSQEFGTSRKKNNGRN    180
PGABG01_0300800-t36_1-p1       MNNNFFKSQAYNESEKRDNLRSKIYNEQNDFPKEGYSREYDSKYNSQEFGTSRKKNNGRN    180
PGSY75_0301600-t31_1-p1        MNNNFFKSQAYNESEKRDNLRSKIYNEQNNFPKEGYSREYDSKYNSQEFGTSRKKNNGRN    180
PBLACG01_0301900-t36_1-p1      MNNNLFKSQAYNESEKRDNLRDNIYNEKNDFAKEGYSREYDSKCNSPEFGTSRKKNDGRN    180
PRG01_0304500-t36_1-p1         MNDGLFKSQAYNESEKRDNLRNNIYNEQNDFAKEGYSREYDSKFNSAEFGTSRKKNGGRN    180
PRCDC_0300700.1-p1             MNDGLFKSQAYNESEKRDNLRNNIYNEQNDFAKEGYSREYDSKFNSAEFGTSRKKNGGRN    180
PfML01_030006900-t41_1-p1      MNDGLFKSQAYNESEKRDNLRNNIYNEQNDFAKEGYSREYDSKYNSAEFGTSRKKNDGRN    180
PfGB4_030007500-t41_1-p1       MNDGLFKSQAYNESEKRDNLRNNIYNEQNDFAKEGYSREYDSKYNSAEFGTSRKKNDGRN    180
PfSD01_030006800-t41_1-p1      MNDGLFKSQAYNESEKRDNLRNNIYNEQNDFAKEGYSREYDSKYNSAEFGTSRKKNDGRN    180
PfKH02_030007200-t41_1-p1      MNDGLFKSQAYNESEKRDNLRNNIYNEQNDFAKEGYSREYDSKYNSAEFGTSRKKNDGKN    180
PfIT_030006400-t41_1-p1        MNDGLFKSQAYNESEKRDNLRNNIYNEQNDFAKEGYSREYDSKYNSAEFGTSRKKNDGKN    180
PfKH01_030006400-t41_1-p1      MNDGLFKSQAYNESEKRDNLRNNIYNEQNDFAKEGYSREYDSKYNSAEFGTSRKKNDGKN    180
PfNF135_030006800.1-p1         MNDGLFKSQAYNESEKRDNLRNNIYNEQNDFAKEGYSREYDSKYNSAEFGTSRKKNDGKN    180
PfCD01_030007000-t41_1-p1      MNDGLFKSQAYNESEKRDNLRNNIYNEQNDFAKEGYSREYDSKYNSAEFGTSRKKNDGRN    180
PfGN01_030007300-t41_1-p1      MNDGLFKSQAYNESEKRDNLRNNIYNEQNDFAKEGYSREYDSKYNSAEFGTSRKKNDGRN    180
PfKE01_030006300-t41_1-p1      MNDGLFKSQAYNESEKRDNLRNNIYNEQNDFAKEGYSREYDSKYNSAEFGTSRKKNDGRN    180
PfSN01_030007100-t41_1-p1      MNDGLFKSQAYNESEKRDNLRNNIYNEQNDFAKEGYSREYDSKYNSAEFGTSRKKNDGRN    180
PfTG01_030008300-t41_1-p1      MNDGLFKSQAYNESEKRDNLRNNIYNEQNDFAKEGYSREYDSKYNSAEFGTSRKKNDGRN    180
Pf7G8-2_000071200.1-p1         MNDGLFKSQAYNESEKRDNLRNNIYNEQNDFAKEGYSREYDSKYNSAEFGTSRKKNDGRN    180
Pf7G8_030007400-t41_1-p1       MNDGLFKSQAYNESEKRDNLRNNIYNEQNDFAKEGYSREYDSKYNSAEFGTSRKKNDGRN    180
PfHB3_030005500-t41_1-p1       MNDGLFKSQAYNESEKRDNLRNNIYNEQNDFAKEGYSREYDSKYNSAEFGTSRKKNDGRN    180
PF3D7_0301600.1-p1             MNDGLFKSQAYNESEKRDNLRNNIYNEQNDFAKEGYSREYDSKYNSAEFGTSRKKNDGRN    180
PfNF54_030006800.1-p1          MNDGLFKSQAYNESEKRDNLRNNIYNEQNDFAKEGYSREYDSKYNSAEFGTSRKKNDGRN    180
PfGA01_030008000-t41_1-p1      MNDGLFKSQAYNESEKRDNLRNNIYNEQNDFAKEGYSREYDSKYNSAEFGTSRKKNDGRN    180
PfDd2_030006600-t41_1-p1       MNDGLFKSQAYNESEKRDNLRNNIYNEQNDFAKEGYSREYDSKYNSAEFGTSRKKNDGKN    180
                               **:.:****************.:****:*:* *********** ** *********.*:*

PADL01_0034200-t36_1-p1        SFNYQSNNENNKHSSYMNEDMYNKNYFNQRENIDELNKKMHDDWSNYLNEDYSHGFESNN    240
PGABG01_0300800-t36_1-p1       SFNYQSNNENNKHSFYMNEDMYNKNYFDQRENIDELNKKMHDDWSNFLIEDYSHEFESNN    240
PGSY75_0301600-t31_1-p1        SFNYQSNNENNKHSFYMNEDMYNKNYFDQRENIDELNKKMHDDWSNFLIEDYSHEFESNN    240
PBLACG01_0301900-t36_1-p1      SFNYQSNNENNKPSSYMNEDIYNKNYYEQRENIDEINKKLHDDWSNFLNENYSSEFESNN    240
PRG01_0304500-t36_1-p1         PFNYQSNNENNKPASFMNEDIYNKNYYNERENIDELNKKLHDDWSKFLNEDYSNEFESNN    240
PRCDC_0300700.1-p1             PFNYQSNNENNKPASFMNEDIYNKNYYNERENIDELNKKLHDDWSKFLNEDYSNEFESNN    240
PfML01_030006900-t41_1-p1      PFNYQSNNENNKPASYMNEDIYNKNYYNQRENIDELNKKLHDDWSNFLNEDYSNEFESNN    240
PfGB4_030007500-t41_1-p1       PFNYQSNNENNKPASYMNEDIYNKNYYNQRENIDELNKKLHDDWSNFLNEDYSNEFESNN    240
PfSD01_030006800-t41_1-p1      PFNYQSNNENNKPASYMNEDIYNKNYYNQRENIDELNKKLHDDWSNFLNEDYSNEFESNN    240
PfKH02_030007200-t41_1-p1      PFNYQSNNENNKPASYMNEDIYNKNYYNQRENIDELNKKLHDDWSNFLNEDYSNEFESNN    240
PfIT_030006400-t41_1-p1        PFNYQSNNENNKPASYMNEDIYNKNYYNQRENIDELNKKLHDDWSNFLNEDYSNEFESNN    240
PfKH01_030006400-t41_1-p1      PFNYQSNNENNKPASYMNEDIYNKNYYNQRENIDELNKKLHDDWSNFLNEDYSNEFESNN    240
PfNF135_030006800.1-p1         PFNYQSNNENNKPASYMNEDIYNKNYYNQRENIDELNKKLHDDWSNFLNEDYSNEFESNN    240
PfCD01_030007000-t41_1-p1      PFNYQSNNENNKPASYMNEDIYNKNYYNQRENIDELNKKLHDDWSNFLNEDYSNEFESNN    240
PfGN01_030007300-t41_1-p1      PFNYQSNNENNKPASYMNEDIYNKNYYNQRENIDELNKKLHDDWSNFLNEDYSNEFESNN    240
PfKE01_030006300-t41_1-p1      PFNYQSNNENNKPASYMNEDIYNKNYYNQRENIDELNKKLHDDWSNFLNEDYSNEFESNN    240
PfSN01_030007100-t41_1-p1      PFNYQSNNENNKPASYMNEDIYNKNYYNQRENIDELNKKLHDDWSNFLNEDYSNEFESNN    240
PfTG01_030008300-t41_1-p1      PFNYQSNNENNKPASYMNEDIYNKNYYNQRENIDELNKKLHDDWSNFLNEDYSNEFESNN    240
Pf7G8-2_000071200.1-p1         PFNYQSNNENNKPASYMNEDIYNKNYYNQRENIDELNKKLHDDWSNFLNEDYSNEFESNN    240
Pf7G8_030007400-t41_1-p1       PFNYQSNNENNKPASYMNEDIYNKNYYNQRENIDELNKKLHDDWSNFLNEDYSNEFESNN    240
PfHB3_030005500-t41_1-p1       PFNYQSNNENNKPASYMNEDIYNKNYYNQRENIDELNKKLHDDWSNFLNEDYSNEFESNN    240
PF3D7_0301600.1-p1             PFNYQSNNENNKPASYMNEDIYNKNYYNQRENIDELNKKLHDDWSNFLNEDYSNEFESNN    240
PfNF54_030006800.1-p1          PFNYQSNNENNKPASYMNEDIYNKNYYNQRENIDELNKKLHDDWSNFLNEDYSNEFESNN    240
PfGA01_030008000-t41_1-p1      PFNYQSNNENNKPASYMNEDIYNKNYYNQRENIDELNKKLHDDWSNFLNEDYSNEFESNN    240
PfDd2_030006600-t41_1-p1       PFNYQSNNENNKPASYMNEDIYNKNYYNQRENIDELNKKLHDDWSNFLNEDYSNEFESNN    240
                                *********** : :****:*****:::******:***:*****::* *:**  *****
```

```
PADL01_0034200-t36_1-p1         FNSPHYDTYKSQYDTYKSQYDTYKSQYGIYNSQYDTYNSQYDDRNNYSYPSSEENYVPPF    300
PGABG01_0300800-t36_1-p1        FNSPHYDTYKSQYDTYKSQYDTYKSQYGIYNSEYDTYNSEYDDRNNYFYPSSEENFVPPF    300
PGSY75_0301600-t31_1-p1         FNSPHYDTYKSQYDTYKSQYDTYKSQYGIYNSEYDTYNSEYDDRNNYFYPSSEENFVPPF    300
PBLACG01_0301900-t36_1-p1       FNSPHYDTYKSQYDTYKSQYDTYKSQYDNYKSQYETYKSQYDDRNKYSYPSRDENYVPPF    300
PRG01_0304500-t36_1-p1          FNSPHYDTYKSQYDTYKSKYDTYKSPYDTYKSQYDIYKSQYDDRNKYSYPSREANYVPPF    300
PRCDC_0300700.1-p1              FNSPHYDTYKSQYDTYKSKYDTYKSPYDTYKSQYDIYKSQYDDRNKYSYPSREANYVPPF    300
PfML01_030006900-t41_1-p1       FNSPHYDTYKSEYDTYKSEYDTYKSQYDTYKSQYDTYKSQYDDRNKYSYPSREGNYVPPF    300
PfGB4_030007500-t41_1-p1        FNSPHYDTYKSEYDTYKSEYDTYKSQYDTYKSQYDTYKSQYDDRNKYSYPSREGNYVPPF    300
PfSD01_030006800-t41_1-p1       FNSPHYDTYKSEYDTYKSEYDTYKSQYDTYKSQYDTYKSQYDDRNKYSYPSREGNYVPPF    300
PfKH02_030007200-t41_1-p1       FNSPHYDTYKSEYDTYKSEYDT-------YKSQYDTYKSQYDDRNKYSYPSREGNYVPPF    293
PfIT_030006400-t41_1-p1         FNSPHYDTYKSEYDTYKSEYDT-------YKSQYDTYKSQYDDRNKYSYPSREGNYVPPF    293
PfKH01_030006400-t41_1-p1       FNSPHYDTYKSEYDTYKSEYDT-------YKSQYDTYKSQYDDRNKYSYPSREGNYVPPF    293
PfNF135_030006800.1-p1          FNSPHYDTYKSEYDTYKSEYDT-------YKSQYDTYKSQYDDRNKYSYPSREGNYVPPF    293
PfCD01_030007000-t41_1-p1       FNSPHYDTYKSEYDTYKSEYDT-------YKSQYDTYKSQYDDRNKYSYPSREGNYVPPF    293
PfGN01_030007300-t41_1-p1       FNSPHYDTYKSEYDTYKSEYDT-------YKSQYDTYKSQYDDRNKYSYPSREGNYVPPF    293
PfKE01_030006300-t41_1-p1       FNSPHYDTYKSEYDTYKSEYDT-------YKSQYDTYKSQYDDRNKYSYPSREGNYVPPF    293
PfSN01_030007100-t41_1-p1       FNSPHYDTYKSEYDTYKSEYDT-------YKSQYDTYKSQYDDRNKYSYPSREGNYVPPF    293
PfTG01_030008300-t41_1-p1       FNSPHYDTYKSEYDTYKSEYDT-------YKSQYDTYKSQYDDRNKYSYPSREGNYVPPF    293
Pf7G8-2_000071200.1-p1          FNSPHYDTYKSEYDTYKSEYDT-------YKSQYDTYKSQYDDRNKYSYPSREGNYVPPF    293
Pf7G8_030007400-t41_1-p1        FNSPHYDTYKSEYDTYKSEYDT-------YKSQYDTYKSQYDDRNKYSYPSREGNYVPPF    293
PfHB3_030005500-t41_1-p1        FNSPHYDTYKSEYDTYKSEYDT-------YKSQYDTYKSQYDDRNKYSYPSREGNYVPPF    293
PF3D7_0301600.1-p1              FNSPHYDTYKSEYDTYKSEYDT-------YKSQYDTYKSQYDDRNKYSYPSREGNYVPPF    293
PfNF54_030006800.1-p1           FNSPHYDTYKSEYDTYKSEYDT-------YKSQYDTYKSQYDDRNKYSYPSREGNYVPPF    293
PfGA01_030008000-t41_1-p1       FNSPHYDTYKSEYDTYKSEYDT-------YKSQYDTYKSQYDDRNKYSYPSREGNYVPPF    293
PfDd2_030006600-t41_1-p1        FNSPHYDTYKSEYDTYKSEYDT-------YKSQYDTYKSQYDDRNKYSYPSREGNYVPPF    293
                                ***********:******:***       *:*:*: *:*:*****:* *** : *:****

PADL01_0034200-t36_1-p1         MDENMNGLNNNYRTGFDESPKNNINDDEGINFVRDEFYDEYDN-NMNTPPQYMFSPEWDN    359
PGABG01_0300800-t36_1-p1        MDENMNGLNNNYRTGFDESQKNNINDDVGINFVRDEFYDEYNN-NMNTPPQYMFSPEWDN    359
PGSY75_0301600-t31_1-p1         MDENMNGLNNNYRTGFDESQKNNINDDEGINFVRDEFYDEYNN-NMNTPPQYMFSPEWDN    359
PBLACG01_0301900-t36_1-p1       MDENMNRLNNNYRSGFDDSQKNNINDDGGMNFVRDEFYDEYNN-NMNTPPQYMFSPEWDN    359
PRG01_0304500-t36_1-p1          MDENINRLNNNYRDGFDDTKKNNINDDKGMNFVRDEFYDEYNN-NMNTSPEYLFSQEWDN    359
PRCDC_0300700.1-p1              MDENINRLNNNYRDGFDDTKKNNINDDKGMNFVRDEFYDEYNN-NMNTSPEYLFSQEWDN    359
PfML01_030006900-t41_1-p1       MDENDNRLNNNYRSGFDDTKKNNINDDKGMNFVRDEFYDEYNNNNMNTSPEYMFSQEWDN    360
PfGB4_030007500-t41_1-p1        MDENVNRLNNNYKSGFDDTKKNNINDDKGMNFVRDEFYDEYNNNNMNTSPEYMFSQEWDN    360
PfSD01_030006800-t41_1-p1       MDENDNRLNNNYRSGFDDTKKNNINDDKGMNFVRDEFYDEYNNNNMNTSPEYMFSQEWDN    360
PfKH02_030007200-t41_1-p1       MDENVNRLNNNYKSGFDDTKKNNINDDKGMNFVRDEFYDEYNNNNMNTSPEYMFSQEWDN    353
PfIT_030006400-t41_1-p1         MDENDNRLNNNYRSGFDDTKKNNINDDKGMNFVRDEFYDEYNNNNMNTSPEYMFSQEWDN    353
PfKH01_030006400-t41_1-p1       MDENDNRLNNNYRSGFDDTKKNNINDDKGMNFVRDEFYDEYNNNNMNTSPEYMFSQEWDN    353
PfNF135_030006800.1-p1          MDENDNRLNNNYRSGFDDTKKNNINDDKGMNFVRDEFYDEYNNNNMNTSPEYMFSQEWDN    353
PfCD01_030007000-t41_1-p1       MDENDNRLNNNYRSGFDDTKKNNINDDKGMNFVRDEFYDEYNNNNMNTSPEYMFSQEWDN    353
PfGN01_030007300-t41_1-p1       MDENDNRLNNNYRSGFDDTKKNNINDDKGMNFVRDEFYDEYNNNNMNTSPEYMFSQEWDN    353
PfKE01_030006300-t41_1-p1       MDENDNRLNNNYRSGFDDTKKNNINDDKGMNFVRDEFYDEYNNNNMNTSPEYMFSQEWDN    353
PfSN01_030007100-t41_1-p1       MDENDNRLNNNYRSGFDDTKKNNINDDKGMNFVRDEFYDEYNNNNMNTSPEYMFSQEWDN    353
PfTG01_030008300-t41_1-p1       MDENDNRLNNNYRSGFDDTKKNNINDDKGMNFVRDEFYDEYNNNNMNTSPEYMFSQEWDN    353
Pf7G8-2_000071200.1-p1          MDENVNRLNNNYKSGFDDTKKNNINDDKGMNFVRDEFYDEYNNNNMNTSPEYMFSQEWDN    353
Pf7G8_030007400-t41_1-p1        MDENVNRLNNNYKSGFDDTKKNNINDDKGMNFVRDEFYDEYNNNNMNTSPEYMFSQEWDN    353
PfHB3_030005500-t41_1-p1        MDENVNRLNNNYKSGFDDTKKNNINDDKGMNFVRDEFYDEYNNNNMNTSPEYMFSQEWDN    353
PF3D7_0301600.1-p1              MDENDNRLNNNYRSGFDDTKKNNINDDKGMNFVRDEFYDEYNNNNMNTSPEYMFSQEWDN    353
PfNF54_030006800.1-p1           MDENDNRLNNNYRSGFDDTKKNNINDDKGMNFVRDEFYDEYNNNNMNTSPEYMFSQEWDN    353
PfGA01_030008000-t41_1-p1       MDENDNRLNNNYRSGFDDTKKNNINDDKGMNFVRDEFYDEYNNNNMNTSPEYMFSQEWDN    353
PfDd2_030006600-t41_1-p1        MDENDNRLNNNYRSGFDDTKKNNINDDKGMNFVRDEFYDEYNNNNMNTSPEYMFSQEWDN    353
                                **** * *****: ***:: ******* *:***********:* **** *:*:** ****
```

```
PADL01_0034200-t36_1-p1        QNTYDGFESGWNNYNSEYNDFGDQVKVETPYIRVVEEINDESDNIRDDYDMLNNNSSCNR    419
PGABG01_0300800-t36_1-p1       QNTYDGFESGWNNYNSEYNDFVDQVNVETPYIRVVEEINDESDNIRDDYDMLNNDSPCNR    419
PGSY75_0301600-t31_1-p1        QNTYDGFESGWNNYNSEYNDFVDQVNVETPYIRVVEEINDESDNIRDDYDMLNNDSPCNR    419
PBLACG01_0301900-t36_1-p1      QNTYDGFTSGWDNYNSTNSDHRDQVKVETPYIRVVEEIDDESNNIRDDFDILNKDSSCNR    419
PRG01_0304500-t36_1-p1         ENTHDGFSSGWNNYNSENSDHKDQIKVETPYIRVVEEIDDESNNKRDNYDMLNKDSSCNR    419
PRCDC_0300700.1-p1             ENTHDGFSSGWNNYNSENSDHKDQIKVETPYIRVVEEIDDESNNKRDNYDMLNKDSSCNR    419
PfML01_030006900-t41_1-p1      QNTHDGFPSGWNNYNSENSDHKDQIKVETPYIRVVEEVNDESNNKRDNYDTLNKDSSCNR    420
PfGB4_030007500-t41_1-p1       QNTHDGFPSGWNNYNSENSDHKDQIKVETPYIRVVEEVNDESNNKRDNYDTLNKDSSCNR    420
PfSD01_030006800-t41_1-p1      QNTHDGFPSGWNNYNSENSDHKDQIKVETPYIRVVEEVNDESNNKRDNYDTLNKDSSCNR    420
PfKH02_030007200-t41_1-p1      QNTHDGFPSGWNNYNSENSDHKDQIKVETPYIRVVEEVNDESNNKRDNYDTLNKDSSCNR    413
PfIT_030006400-t41_1-p1        QNTHDGFPSGWNNYNSENSDHKDQIKVETPYIRVVEEVNDESNNKRDNYDTLNKDSSCNR    413
PfKH01_030006400-t41_1-p1      QNTHDGFPSGWNNYNSENSDHKDQIKVETPYIRVVEEVNDESNNKRDNYDTLNKDSSCNR    413
PfNF135_030006800.1-p1         QNTHDGFPSGWNNYNSENSDHKDQIKVETPYIRVVEEVDDESNNKRDNYDTLNKDSSCNR    413
PfCD01_030007000-t41_1-p1      QNTHDGFPSGWNNYNSENSDHKDQIKVETPYIRVVEEVNDESNNKRDNYDTLNKDSSCNR    413
PfGN01_030007300-t41_1-p1      QNTHDGFPSGWNNYNSENSDHKDQIKVETPYIRVVEEVNDESNNKRDNYDTLNKDSSCNR    413
PfKE01_030006300-t41_1-p1      QNTHDGFPSGWNNYNSENSDHKDQIKVETPYIRVVEEVNDESNNKRDNYDTLNKDSSCNR    413
PfSN01_030007100-t41_1-p1      QNTHDGFPSGWNNYNSENSDHKDQIKVETPYIRVVEEVNDESNNKRDNYDTLNKDSSCNR    413
PfTG01_030008300-t41_1-p1      QNTHDGFPSGWNNYNSENSDHKDQIKVETPYIRVVEEVNDESNNKRDNYDTLNKDSSCNR    413
Pf7G8-2_000071200.1-p1         QNTHDGFPSGWNNYNSENSDHKDQIKVETPYIRVFEEVNDESNNKRDNYDTLNKDSSCNR    413
Pf7G8_030007400-t41_1-p1       QNTHDGFPSGWNNYNSENSDHKDQIKVETPYIRVFEEVNDESNNKRDNYDTLNKDSSCNR    413
PfHB3_030005500-t41_1-p1       QNTHDGFPSGWNNYNSENSDHKDQIKVETPYIRVVEEVNDESNNKRDNYDTLNKDSSCNR    413
PF3D7_0301600.1-p1             QNTHDGFPSGWNNYNSENSDHKDQIKVETPYIRVVEEVNDESNNKRDNYDTLNKDSSCNR    413
PfNF54_030006800.1-p1          QNTHDGFPSGWNNYNSENSDHKDQIKVETPYIRVVEEVNDESNNKRDNYDTLNKDSSCNR    413
PfGA01_030008000-t41_1-p1      QNTHDGFPSGWNNYNSENSDHKDQIKVETPYIRVVEEVNDESNNKRDNYDTLNKDSSCNR    413
PfDd2_030006600-t41_1-p1       QNTHDGFPSGWNNYNSENSDHKDQIKVETPYIRVVEEVNDESNNKRDNYDTLNKDSSCNR    413
                               :**:*** ***:****  .*. **::********.**::***:* **::* **::* ***

PADL01_0034200-t36_1-p1        NEKMEYYFQSDKFPVDCKGIYTNSNIVVEEYDTPIDVEDTNNNVYSCSDRLNNERQKKHM    479
PGABG01_0300800-t36_1-p1       NEKMEYYFQSDKFPVNCKGIYTNSNIVVEEYDIPIDIKDPNNNVYSYNDRLNNERQKKHM    479
PGSY75_0301600-t31_1-p1        NEKMEYYFQSDKFPVNCKGIYTNSNIVVEEYDIPIDIKDPNNNVYSYNDRLNNERQKKHM    479
PBLACG01_0301900-t36_1-p1      NEKMEYYFQSDKFPVDCKGIYKNSNIVVEEYDTPVDLKEPDNNVYSFNERLNNERQKKYM    479
PRG01_0304500-t36_1-p1         NEKMEYYFQSDKFPVDCKGMYTNSNIVVEEYDTPVDLKETNNNFYSFNEKLNNERPKKYM    479
PRCDC_0300700.1-p1             NEKMEYYFQSDKFPVDCKGMYTNSNIVVEEYDTPVDLKETNNNFYSFNEKLNNERPKKYM    479
PfML01_030006900-t41_1-p1      NEKMEYYFQSDKFPVDCKGIYTNSNIIVEEYDTPVDLKETNDNFYSFNEKLNNERQKKYM    480
PfGB4_030007500-t41_1-p1       NEKMEYYFQSDKFPVDCKGIYTNSNIIVEEYDTPVDLKETNDNFYSFNEKLNNERQKKYM    480
PfSD01_030006800-t41_1-p1      NEKMEYYFQSDKFPVDCKGIYTNSNIIVEEYDTPVDLKETNDNFYSFNEKLNNERQKKYM    480
PfKH02_030007200-t41_1-p1      NEKMEYYFQSDKFPVDCKGIYTNSNIIVEEYDTPVDLKETNDNFYSFNEKLNNERQKKYM    473
PfIT_030006400-t41_1-p1        NEKMEYYFQSDKFPVDCKGIYTNSNIIVEEYDTPVDLKETNDNFYSFNEKLNNERQKKYM    473
PfKH01_030006400-t41_1-p1      NEKMEYYFQSDKFPVDCKGIYTNSNIIVEEYDTPVDLKETNDNFYSFNEKLNNERQKKYM    473
PfNF135_030006800.1-p1         NEKMEYYFQSDKFPVDCKGIYTNSNIIVEEYDTPVDLKETNDNFYSFNEKLNNERQKKYM    473
PfCD01_030007000-t41_1-p1      NEKMEYYFQSDKFPVDCKGIYTNSNIIVEEYDTPVDLKETNDNFYSFNEKLNNERQKKYM    473
PfGN01_030007300-t41_1-p1      NEKMEYYFQSDKFPVDCKGIYTNSNIIVEEYDTPVDLKETNDNFYSFNEKLNNERQKKYM    473
PfKE01_030006300-t41_1-p1      NEKMEYYFQSDKFPVDCKGIYTNSNIIVEEYDTPVDLKETNDNFYSFNEKLNNERQKKYM    473
PfSN01_030007100-t41_1-p1      NEKMEYYFQSDKFPVDCKGIYTNSNIIVEEYDTPVDLKETNDNFYSFNEKLNNERQKKYM    473
PfTG01_030008300-t41_1-p1      NEKMEYYFQSDKFPVDCKGIYTNSNIIVEEYDTPVDLKETNDNFYSFNEKLNNERQKKYM    473
Pf7G8-2_000071200.1-p1         NEKMEYYFQSDKFPVDCKGIYTNSNIIVEEYDTPVDLKETNDNFYSFNEKLNNERQKKYM    473
Pf7G8_030007400-t41_1-p1       NEKMEYYFQSDKFPVDCKGIYTNSNIIVEEYDTPVDLKETNDNFYSFNEKLNNERQKKYM    473
PfHB3_030005500-t41_1-p1       NEKMEYYFQSDKFPVDCKGIYTNSNIIVEEYDTPVDLKETNDNFYSFNEKLNNERQKKYM    473
PF3D7_0301600.1-p1             NEKMEYYFQSDKFPVDCKGIYTNSNIIVEEYDTPVDLKETNDNFYSFNEKLNNERQKKYM    473
PfNF54_030006800.1-p1          NEKMEYYFQSDKFPVDCKGIYTNSNIIVEEYDTPVDLKETNDNFYSFNEKLNNERQKKYM    473
PfGA01_030008000-t41_1-p1      NEKMEYYFQSDKFPVDCKGIYTNSNIIVEEYDTPVDLKETNDNFYSFNEKLNNERQKKYM    473
PfDd2_030006600-t41_1-p1       NEKMEYYFQSDKFPVDCKGIYTNSNIIVEEYDTPVDLKETNDNFYSFNEKLNNERQKKYM    473
                               ***************:***:*.****:***** *:*::: ::*.** .::***** **:*
```

```
PADL01_0034200-t36_1-p1         QEDKEFVNEKFKNDIGHTPSSNSS--------------------------------SSHS    507
PGABG01_0300800-t36_1-p1        QEDKEFVNEKFKNDIGYTPSSNSS--------------------------------SPHS    507
PGSY75_0301600-t31_1-p1         QQDKEFVNEKFKNDIGYTPSSNSS--------------------------------FPHS    507
PBLACG01_0301900-t36_1-p1       QEDKEFKNEKFKNDIGHMSSPSSTLSNSSS-----------PHMPSSH-----------S    517
PRG01_0304500-t36_1-p1          PEDKEFIN-----EIGHMSSTWSNSSSSHSPSSHFSSSHSSSSHSPSSHSPSSHFSSSHS    534
PRCDC_0300700.1-p1              PEDKEFIN-----EIGHMSSTWSNSSSSHSSSSHSS-----SSHFASSHSSSSHFSS---    526
PfML01_030006900-t41_1-p1       PEDKEFIN-----EMGHMSSTS--------------------------------------    497
PfGB4_030007500-t41_1-p1        PEDKEFIN-----EMGHMSSTS--------------------------------------    497
PfSD01_030006800-t41_1-p1       PEDKEFIN-----EMGHMSSTS--------------------------------------    497
PfKH02_030007200-t41_1-p1       PEDKEFIN-----EMGHMSSTS--------------------------------------    490
PfIT_030006400-t41_1-p1         PEDKEFIN-----EMGHMSSTS--------------------------------------    490
PfKH01_030006400-t41_1-p1       PEDKEFIN-----EMGHMSSTS--------------------------------------    490
PfNF135_030006800.1-p1          PEDKEFIN-----EMGHMSSTS--------------------------------------    490
PfCD01_030007000-t41_1-p1       PEDKEFIN-----EMGHMSSTS--------------------------------------    490
PfGN01_030007300-t41_1-p1       PEDKEFIN-----EMGHMSSTS--------------------------------------    490
PfKE01_030006300-t41_1-p1       PEDKEFIN-----EMGHMSSTS--------------------------------------    490
PfSN01_030007100-t41_1-p1       PEDKEFIN-----EMGHMSSTS--------------------------------------    490
PfTG01_030008300-t41_1-p1       PEDKEFIN-----EMGHMSSTS--------------------------------------    490
Pf7G8-2_000071200.1-p1          PEDKEFIN-----EMGHMSSTS--------------------------------------    490
Pf7G8_030007400-t41_1-p1        PEDKEFIN-----EMGHMSSTS--------------------------------------    490
PfHB3_030005500-t41_1-p1        PEDKEFIN-----EMGHMSSTS--------------------------------------    490
PF3D7_0301600.1-p1              REDKEFIN-----EMGHMSSTS--------------------------------------    490
PfNF54_030006800.1-p1           REDKEFIN-----EMGHMSSTS--------------------------------------    490
PfGA01_030008000-t41_1-p1       PEDKEFIN-----EMGHMSSTS--------------------------------------    490
PfDd2_030006600-t41_1-p1        PEDKEFIN-----EMGHMSSTS--------------------------------------    490
                                 :**** *     ::*:  *

PADL01_0034200-t36_1-p1         QPMFTP------------------------------------------ETFQTSNIESPE    525
PGABG01_0300800-t36_1-p1        QPMFTP------------------------------------------ETFQTSNIESPE    525
PGSY75_0301600-t31_1-p1         QPMFTP------------------------------------------ETFQTSNIESPE    525
PBLACG01_0301900-t36_1-p1       QPSFTPETFQTSNIESSEFEEPNIESPEFEEPNIESPEFEEPNI--ESPEFETSNIDSPN    575
PRG01_0304500-t36_1-p1          PPSHSSSSHSPPSHSSSSH-SPPSHSPPSHSSSSHSPPSHSPSSHFPSPH-SQPSFIPET    592
PRCDC_0300700.1-p1              -------SHSPPSHSSSSH-SPPSHSPP-----SHSPPSHSPSSHFPSPH-SQPSFIPET    572
PfML01_030006900-t41_1-p1       --------------------------------------SNSPSSHFPSPH-SQPSFIPET    518
PfGB4_030007500-t41_1-p1        --------------------------------------SNSPSSHFPSPH-SQPSFIPET    518
PfSD01_030006800-t41_1-p1       --------------------------------------SNSPSSHFPSPH-SQPSFIPET    518
PfKH02_030007200-t41_1-p1       --------------------------------------SNSPSSHFPSPH-SQPSFIPET    511
PfIT_030006400-t41_1-p1         --------------------------------------SNSPSSHFPSPH-SQPSFIPET    511
PfKH01_030006400-t41_1-p1       --------------------------------------SNSPSSHFPSPH-SQPSFIPET    511
PfNF135_030006800.1-p1          --------------------------------------SNSPSSHFPSPH-SQPSFIPET    511
PfCD01_030007000-t41_1-p1       --------------------------------------SNSPSSHFPSPH-SQPSFIPET    511
PfGN01_030007300-t41_1-p1       --------------------------------------SNSPSSHFPSPH-SQPSFIPET    511
PfKE01_030006300-t41_1-p1       --------------------------------------SNSPSSHFPSPH-SQPSFIPET    511
PfSN01_030007100-t41_1-p1       --------------------------------------SNSPSSHFPSPH-SQPSFIPET    511
PfTG01_030008300-t41_1-p1       --------------------------------------SNSPSSHFPSPH-SQPSFIPET    511
Pf7G8-2_000071200.1-p1          --------------------------------------SNSPSSHFPSPH-SQPSFIPET    511
Pf7G8_030007400-t41_1-p1        --------------------------------------SNSPSSHFPSPH-SQPSFIPET    511
PfHB3_030005500-t41_1-p1        --------------------------------------SNSPSSHFPSPH-SQPSFIPET    511
PF3D7_0301600.1-p1              --------------------------------------SNSPSSHFPSPH-SQPSFIPET    511
PfNF54_030006800.1-p1           --------------------------------------SNSPSSHFPSPH-SQPSFIPET    511
PfGA01_030008000-t41_1-p1       --------------------------------------SNSPSSHFPSPH-SQPSFIPET    511
PfDd2_030006600-t41_1-p1        --------------------------------------SNSPSSHFPSPH-SQPSFIPET    511
                                                                                   .  .:
```

```
PADL01_0034200-t36_1-p1        FQTP----------TFESPPVNTSDYEIPHFNLSEYDL--------------------P-     554
PGABG01_0300800-t36_1-p1       FQTPTFESPEFQIPTFESPPVNTSDYEIPHFNLSEYDLPHLDPSEYDLPHFDPSEYDLP-     584
PGSY75_0301600-t31_1-p1        FQTPTFESPEFQIPTFESPPVNTSDYEIPHFNLSEYDL--------------------P-     564
PBLACG01_0301900-t36_1-p1      FQAPSFESRAFQLPSFESPPVLTSEYEIPNFDLSEYDLPHLDPSEFKPD-----------     624
PRG01_0304500-t36_1-p1         FQESSIESPEFQLPTFESAPVHISEYELPNFDLLEYDLPHFDPSEFRPEIFDESEFKTEI     652
PRCDC_0300700.1-p1             FQESNVESPEFQLPTFESAPVHISEYELPNFDLLEYDLPHFDPSEFRPEIFDESEFKTEI     632
PfML01_030006900-t41_1-p1      FQDSNIESPEFELPTFESATVHTSEYELPNFDILEYDLPHFDPSEFKPEIFDESEL----     574
PfGB4_030007500-t41_1-p1       FQDSNIESPEFELPTFESATVHTSEYELPNFDILEYDLPHFDPSEFKPEIF---------     569
PfSD01_030006800-t41_1-p1      FQDSNIESPEFELPTFESATVHTSEYELPNFDILEYDLPHFDPSEFKPEIF---------     569
PfKH02_030007200-t41_1-p1      FQDSNIESPEFELPTFESATVHTSEYELPNFDILEYDLPHFDPSEFKPEIFDESEF----     567
PfIT_030006400-t41_1-p1        FQDSNIESPEFELPTFESATVHTSEYELPNFDILEYDLPHFDPSEFKPEIFD--------     563
PfKH01_030006400-t41_1-p1      FQDSNIESPEFELPTFESATVHTSEYELPNFDILEYDLPHFDPSEFKPEIFD--------     563
PfNF135_030006800.1-p1         FQDSNIESPEFELPTFESATVHTSEYELPNFDILEYDLPHFDPSEFKPEIFD--------     563
PfCD01_030007000-t41_1-p1      FQDSNIESPEFELPTFESATVHTSEYELPNFDILEYDLPHFDPSEFKPEIFD--------     563
PfGN01_030007300-t41_1-p1      FQDSNIESPEFELPTFESATVHTSEYELPNFDILEYDLPHFDPSEFKPEIFD--------     563
PfKE01_030006300-t41_1-p1      FQDSNIESPEFELPTFESATVHTSEYELPNFDILEYDLPHFDPSEFKPEIFD--------     563
PfSN01_030007100-t41_1-p1      FQDSNIESPEFELPTFESATVHTSEYELPNFDILEYDLPHFDPSEFKPEIFD--------     563
PfTG01_030008300-t41_1-p1      FQDSNIESPEFELPTFESATVHTSEYELPNFDILEYDLPHFDPSEFKPEIFD--------     563
Pf7G8-2_000071200.1-p1         FQDSNIESPEFELPTFESATVHTSEYELPNFDILEYDLPHFDPSEFKPEIFD--------     563
Pf7G8_030007400-t41_1-p1       FQDSNIESPEFELPTFESATVHTSEYELPNFDILEYDLPHFDPSEFKPEIFD--------     563
PfHB3_030005500-t41_1-p1       FQDSNIESPEFELPTFESATVHTSEYELPNFDILEYDLPHFDPSEFKPEIFD--------     563
PF3D7_0301600.1-p1             FQDSNIESPEFELPTFESATVHTSEYELPNFDILEYDLPHFDPSEFKPEIFDESEF----     567
PfNF54_030006800.1-p1          FQDSNIESPEFELPTFESATVHTSEYELPNFDILEYDLPHFDPSEFKPEIFDESEF----     567
PfGA01_030008000-t41_1-p1      FQDSNIESPEFELPTFESATVHTSEYELPNFDILEYDLPHFDPSEFKPEIFDESEF----     567
PfDd2_030006600-t41_1-p1       FQDSNIESPEFELPTFESATVHTSEYELPNFDILEYDLPHFDPSEFKPEIFDESEF----     567
                               **            :***  *  *:**:*:*:: ****

PADL01_0034200-t36_1-p1        ---------HLDPSEFKPEPFDESEFDPPLFDPSEYKNSSFKQQKYSASSNSSRSYENEF     605
PGABG01_0300800-t36_1-p1       ---------HLDPSEFKPEPFDESEFDPPVFDPSEYKNSTFKQPKYSTSSHSSRSYENEF     635
PGSY75_0301600-t31_1-p1        ---------HLDPSEFKPEPFDESEFDPPVFDPSEYKNSTFKQPKYSTSSHSSRSYENEF     615
PBLACG01_0301900-t36_1-p1      -------------------PFDESDFKPQVFDPSEYKNSTLKQSKLSTSSNSAYSYENEF     665
PRG01_0304500-t36_1-p1         FDESEF----------RPEIFDESEFKPSVFDTSEYKNSTFKESKFSTSSHSTRSYENEF     702
PRCDC_0300700.1-p1             FDESEFRPEIFDESEFRPEIFDESEFKPSVFDPSEYKNSTFKESKFSTSSHSTRSYENEF     692
PfML01_030006900-t41_1-p1      ------K----------PEIFDESEFKPSVFDPSEYKNSPFKESKISTSSHSTRSYENEF     618
PfGB4_030007500-t41_1-p1       ---------------------DESEFKPSVFDPSEYKNSPFKESKISTSSHSTRSYENEF     608
PfSD01_030006800-t41_1-p1      ---------------------DESEFKPSVFDPSEYKNSPFKESKISTSSHSTRSYENEF     608
PfKH02_030007200-t41_1-p1      ------KPEIFDESEFKPEIFDESEFKPSVFDPSEYKNSPFKESKISTSSHSTRSYENEF     621
PfIT_030006400-t41_1-p1        ----------------------ESEFKPSVFDPSEYKNSPFKESKISTSSHSTRSYENEF     601
PfKH01_030006400-t41_1-p1      ----------------------ESEFKPSVFDPSEYKNSPFKESKISTSSHSTRSYENEF     601
PfNF135_030006800.1-p1         ----------------------ESEFKPSVFDPSEYKNSPFKESKISTSSHSTRSYENEF     601
PfCD01_030007000-t41_1-p1      ----------------------ESEFKPSVFDPSEYKNSPFKESKISTSSHSTRSYENEF     601
PfGN01_030007300-t41_1-p1      ----------------------ESEFKPSVFDPSEYKNSPFKESKISTSSHSTRSYENEF     601
PfKE01_030006300-t41_1-p1      ----------------------ESEFKPSVFDPSEYKNSPFKESKISTSSHSTRSYENEF     601
PfSN01_030007100-t41_1-p1      ----------------------ESEFKPSVFDPSEYKNSPFKESKISTSSHSTRSYENEF     601
PfTG01_030008300-t41_1-p1      ----------------------ESEFKPSVFDPSEYKNSPFKESKISTSSHSTRSYENEF     601
Pf7G8-2_000071200.1-p1         ----------------------ESEFKPSVFDPSEYKNSPFKESKISTSSHSTRSYENEF     601
Pf7G8_030007400-t41_1-p1       ----------------------ESEFKPSVFDPSEYKNSPFKESKISTSSHSTRSYENEF     601
PfHB3_030005500-t41_1-p1       ----------------------ESEFKPSVFDPSEYKNSPFKESKISTSSHSTRSYENEF     601
PF3D7_0301600.1-p1             ------K----------PEIFDESEFKPSVFDPSEYKNSPFKESKISTSSHSTRSYENEF     611
PfNF54_030006800.1-p1          ------K----------PEIFDESEFKPSVFDPSEYKNSPFKESKISTSSHSTRSYENEF     611
PfGA01_030008000-t41_1-p1      ------K----------PEIFDESEFKPSVFDPSEYKNSPFKESKISTSSHSTRSYENEF     611
PfDd2_030006600-t41_1-p1       ------K----------PEIFDESEFKPSVFDPSEYKNSPFKESKISTSSHSTRSYENEF     611
                                                     **:*.* :** ****** :*: * *:**:*: ******
```

```
PADL01_0034200-t36_1-p1        NLSTHRRNCESSSRIHVDDENYKRLKNILSSLCSSREKIYDLSKEEKEFLVKMLKLDYED    665
PGABG01_0300800-t36_1-p1       NLSTHRRNCESSSRIHVDDENYMRLKNILSSLCSSREKIYDLSKEEKEFLVKMLKLDYED    695
PGSY75_0301600-t31_1-p1        NLSTHRRNCESSSRIHVDDENYMRLKNILSSLCSSREKIYDLSKEEKEFLVKMLKLDYED    675
PBLACG01_0301900-t36_1-p1      NLSTHRRNSESSSRIHVDDENYKRLKNILSNLCSSREKIYDLSKEEKEFLVKMLKLDYED    725
PRG01_0304500-t36_1-p1         NLSSDRRNSESSSRIHVDDENYKRLKDILSNLCSSREKIYDLSKEEKEFLVKMLKLDYED    762
PRCDC_0300700.1-p1             NLSSDRRNSESSSRIHVDDENYKRLKDILSNLCSSREKIYDLSKEEKEFLVKMLKLDYED    752
PfML01_030006900-t41_1-p1      NLSTDRRNSESSSRIHVDDENYKRLKKILSNLCSSREKIYDLSKEEKEFLVKMLQLDYED    678
PfGB4_030007500-t41_1-p1       NLSTDRRNSESSSRIHVDDENYKRLKKILSNLCSSREKIYDLSKEEKEFLVKMLQLDYED    668
PfSD01_030006800-t41_1-p1      NLSTDRRNSESSSRIHVDDENYKRLKKILSNLCSSREKIYDLSKEEKEFLVKMLQLDYED    668
PfKH02_030007200-t41_1-p1      NLSTDRRNSESSSRIHVDDENYKRLKKILSNLCSSREKIYDLSKEEKQFLVKMLQLDYED    681
PfIT_030006400-t41_1-p1        NLSTDRRNSESSSRIHVDDENYKRLKKILSNLCSSREKIYDLSKEEKEFLVKMLQLDYED    661
PfKH01_030006400-t41_1-p1      NLSTDRRNSESSSRIHVDDENYKRLKKILSNLCSSREKIYDLSKEEKQFLVKMLQLDYED    661
PfNF135_030006800.1-p1         NLSTDRRNSESSSRIHVDDENYKRLKKILSNLCSSREKIYDLSKEEKEFLVKMLQLDYED    661
PfCD01_030007000-t41_1-p1      NLSTDRRNSESSSRIHVDDENYKRLKKILSNLCSSREKIYDLSKEEKEFLVKMLQLDYED    661
PfGN01_030007300-t41_1-p1      NLSTDRRNSESSSRIHVDDENYKRLKKILSNLCSSREKIYDLSKEEKEFLVKMLQLDYED    661
PfKE01_030006300-t41_1-p1      NLSTDRRNSESSSRIHVDDENYKRLKKILSNLCSSREKIYDLSKEEKEFLVKMLQLDYED    661
PfSN01_030007100-t41_1-p1      NLSTDRRNSESSSRIHVDDENYKRLKKILSNLCSSREKIYDLSKEEKEFLVKMLQLDYED    661
PfTG01_030008300-t41_1-p1      NLSTDRRNSESSSRIHVDDENYKRLKKILSNLCSSREKIYDLSKEEKEFLVKMLQLDYED    661
Pf7G8-2_000071200.1-p1         NLSTDRRNSESSSRIHVDDENYKRLKKILSNLCSSREKIYDLSKEEKEFLVKMLQLDYED    661
Pf7G8_030007400-t41_1-p1       NLSTDRRNSESSSRIHVDDENYKRLKKILSNLCSSREKIYDLSKEEKEFLVKMLQLDYED    661
PfHB3_030005500-t41_1-p1       NLSTDRRNSESSSRIHVDDENYKRLKKILSNLCSSREKIYDLSKEEKEFLVKMLQLDYED    661
PF3D7_0301600.1-p1             NLSTDRRNSESSSRIHVDDENYKRLKKILSNLCSSREKIYDLSKEEKEFLVKMLQLDYED    671
PfNF54_030006800.1-p1          NLSTDRRNSESSSRIHVDDENYKRLKKILSNLCSSREKIYDLSKEEKEFLVKMLQLDYED    671
PfGA01_030008000-t41_1-p1      NLSTDRRNSESSSKIHVDDENYKRLKKILSNLCSSREKIYDLSKEEKEFLVKMLQLDYED    671
PfDd2_030006600-t41_1-p1       NLSTDRRNSESSSRIHVDDENYKRLKKILSNLCSSREKIYDLSKEEKEFLVKMLQLDYED    671
                               ***:.***.****:******** ***.***.****************:******:*****

PADL01_0034200-t36_1-p1        FNFNYRENPRIYNESSSFTNFELKRIHSKIKAFIYKCLLRLLQSYEKSTENHGCANIYDI    725
PGABG01_0300800-t36_1-p1       FNFNSRQNPRIYNESTSFTNFELKRIHSKIKAFIYKCLLRLLQSYEKSSENHGYANIYDI    755
PGSY75_0301600-t31_1-p1        FNFNSRQNARIYNESSSFTNFELKRIHSKIKAFIYKCLLRLLQSYEKSSENHGYANIYDI    735
PBLACG01_0301900-t36_1-p1      FNFNYRGNTRHYNESSPFTNFELKRIHLKVKAFIYKCLLRLLHSCEKSTGNYVDSNIYDI    785
PRG01_0304500-t36_1-p1         FNFNSRGNTRNYNDSSPFTNFELKRIHLKIKAFIYKCLLRLLQSYEKTTENHVGSNIYDI    822
PRCDC_0300700.1-p1             FNFNSRGNTRNYNDSSPFTNFELKRIHLKIKAFIYKCLLRLLQSYEKTTENHVGSNIYDI    812
PfML01_030006900-t41_1-p1      FNFNYRGNTRSYNDSSPFTNFELKRIHLKIKAFIYKCLLRLLQSYEKSTESRVGSNIYDI    738
PfGB4_030007500-t41_1-p1       FNFNYRGNTRSYNDSSPFTNFELKRIHLKIKAFIYKCLLRLLQSYEKSTESRVGSNIYDI    728
PfSD01_030006800-t41_1-p1      FNFNYRGNTRSYNDSSPFTNFELKRIHLKIKEFIYKCLLRLLQSYEKSTESRVGSNIYDI    728
PfKH02_030007200-t41_1-p1      FNFNYRGNTRSYNDSSPFTNFELKRIHLKIKAFIYKCLLRLLQSYEKSTESRVGSNIYDI    741
PfIT_030006400-t41_1-p1        FNFNYRGNTRSYNDSSPFTNFELKRIHLKIKAFIYKCLLRLLQSYEKSTESRVGSNIYDI    721
PfKH01_030006400-t41_1-p1      FNFNYRGNTRSYNDSSPFTNFELKRIHLKIKAFIYKCLLRLLQSYEKSTESRVGSNIYDI    721
PfNF135_030006800.1-p1         FNFNYRGNTRSYNDSSPFTNFELKRIHLKIKAFIYKCLLRLLQSYEKSTESRVGSNIYDI    721
PfCD01_030007000-t41_1-p1      FNFNYRGNTRSYNDSSPFTNFELKRIHLKIKAFIYKCLLRLLQSYEKSTESRVGSNIYDI    721
PfGN01_030007300-t41_1-p1      FNFNYRGNTRSYNDSSPFTNFELKRIHLKIKAFIYKCLLRLLQSYEKSTESRVGSNIYDI    721
PfKE01_030006300-t41_1-p1      FNFNYRGNTRSYNDSSPFTNFELKRIHLKIKAFIYKCLLRLLQSYEKSTESRVGSNIYDI    721
PfSN01_030007100-t41_1-p1      FNFNYRGNTRSYNDSSPFTNFELKRIHLKIKAFIYKCLLRLLQSYEKSTESRVGSNIYDI    721
PfTG01_030008300-t41_1-p1      FNFNYRGNTRSYNDSSPFTNFELKRIHLKIKAFIYKCLLRLLQSYEKSTESRVGSNIYDI    721
Pf7G8-2_000071200.1-p1         FNFNYRGNTRSYNDSSPFTNFELKRIHLKIKAFIYKCLLRLLQSYEKSTESRVGSNIYDI    721
Pf7G8_030007400-t41_1-p1       FNFNYRGNTRSYNDSSPFTNFELKRIHLKIKAFIYKCLLRLLQSYEKSTESRVGSNIYDI    721
PfHB3_030005500-t41_1-p1       FNFNYRGNTRSYNDSSPFTNFELKRIHLKIKAFIYKCLLRLLQSYEKSTESRVGSNIYDI    721
PF3D7_0301600.1-p1             FNFNYRGNTRSYNDSSPFTNFELKRIHLKIKAFIYKCLLRLLQSYEKSTESRVGSNIYDI    731
PfNF54_030006800.1-p1          FNFNYRGNTRSYNDSSPFTNFELKRIHLKIKAFIYKCLLRLLQSYEKSTESRVGSNIYDI    731
PfGA01_030008000-t41_1-p1      FNFNYRGNTRSYNDSSPFTNFELKRIHLKIKAFIYKCLLRLLQSYEKSTESRVGSNIYDI    731
PfDd2_030006600-t41_1-p1       FNFNYRGNTRSYNDSSPFTNFELKRIHLKIKAFIYKCLLRLLQSYEKSTESRVGSNIYDI    731
                               **** * * * **:*: ********** *:* **********:* **:: .   :*****
```

```
PADL01_0034200-t36_1-p1        Y      726
PGABG01_0300800-t36_1-p1       Y      756
PGSY75_0301600-t31_1-p1        Y      736
PBLACG01_0301900-t36_1-p1      Y      786
PRG01_0304500-t36_1-p1         Y      823
PRCDC_0300700.1-p1             Y      813
PfML01_030006900-t41_1-p1      Y      739
PfGB4_030007500-t41_1-p1       Y      729
PfSD01_030006800-t41_1-p1      Y      729
PfKH02_030007200-t41_1-p1      Y      742
PfIT_030006400-t41_1-p1        Y      722
PfKH01_030006400-t41_1-p1      Y      722
PfNF135_030006800.1-p1         Y      722
PfCD01_030007000-t41_1-p1      Y      722
PfGN01_030007300-t41_1-p1      Y      722
PfKE01_030006300-t41_1-p1      Y      722
PfSN01_030007100-t41_1-p1      Y      722
PfTG01_030008300-t41_1-p1      Y      722
Pf7G8-2_000071200.1-p1         Y      722
Pf7G8_030007400-t41_1-p1       Y      722
PfHB3_030005500-t41_1-p1       Y      722
PF3D7_0301600.1-p1             Y      732
PfNF54_030006800.1-p1          Y      732
PfGA01_030008000-t41_1-p1      Y      732
PfDd2_030006600-t41_1-p1       Y      732
                               *
```

Alignment Syntenic Orthologues PF3D7_0113300

```
PADL01_0110900-t36_1-p1        ------MKNIKRELFFFFVLLFFLYIICGCIYENFHECHYNKIFNNPHASEKYRQFNKIR    54
PGABG01_0111700-t36_1-p1       ------MKNIKRELFLFFVLLFFLYIICGCIYEKLYECQYNKIFNNPHESEKYRQFNKIR    54
PGSY75_0113300-t31_1-p1        ------MKNIKRELFLFFVVLFFLYIICGCIYEKLYECQYNKIYNNPHESEKYRQFNKIR    54
SPJ08214.1                     ------MKNIKSEGLIFFLLLFFIYIIFGCIHENLHECSYKKTLKSLHVSEKYREFNKIR    54
PF3D7_0113300.1-p1             MKNIKNMKNIKSEGFIFFVLVFFICIIFGCIYESLHEGPYKKTLNSLHESTKYRHFNKIR    60
PfNF54_010017900.1-p1          MKNIKNMKNIKSEGFIFFVLVFFICIIFGCIYESLHEGPYKKTLNSLHESTKYRHFNKIR    60
PfGB4_010017100-t41_1-p1       MKNIKNMKNIKSEGFIFFVLVFFICIIFGCIYESLHEGPYKKTLNSLHESTKYRHFNKIR    60
SOS76152.1                     MKNIKNMKNIKSEGFIFFVLVFFICIIFGCIYESLHECPYKKTLNSLHESTKYRHFNKIR    60
PfHB3_010017200-t41_1-p1       MKNIKNMKNIKSEGFIFFVLVFFICIIFGCIYESLHEGPYKKTLNSLHESTKYRHFNKIR    60
PfTG01_010018100-t41_1-p1      MKNIKNMKNIKSEGFIFFVLVFFICIIFGCIYESLHEGPYKKTLNSLHESTKYRHFNKIR    60
PfNF135_010017200.1-p1         MKNIKNMKNIKSEGFIFFVLVFFICIIFGCIYESLHEGPYKKTLNSLHESTKYRHFNKIR    60
PfKH02_010016900-t41_1-p1      MKNIKNMKNIKSEGFIFFVLVFFICIIFGCIYESLHEGPYKKTLNSLHESTKYRHFNKIR    60
PfNF166_010016800.1-p1         MKNIKNMKNIKSEGFIFFVLVFFICIIFGCIYESLHEGPYKKTLNSLHESTKYRHFNKIR    60
Pf7G8-2_000046900.1-p1         MKNIKNMKNIKSEGFIFFVLVFFICIIFGCIYESLHEGPYKKTLNSLHESTKYRHFNKIR    60
Pf7G8_010017300-t41_1-p1       MKNIKNMKNIKSEGFIFFVLVFFICIIFGCIYESLHEGPYKKTLNSLHESTKYRHFNKIR    60
PfKE01_010016300-t41_1-p1      MKNIKNMKNIKSEGFIFFVLVFFICIIFGCIYESLHEGPYKKTLNSLHESTKYRHFNKIR    60
PfIT_010016400-t41_1-p1        MKNIKNMKNIKSEGFIFFVLVFFICIIFGCIYESLHEGPYKKTLNSLHESTKYRHFNKIR    60
PfSN01_010015800-t41_1-p1      MKNIKNMKNIKSEGFIFFVLVFFICIIFGCIYESLHEGPYKKTLNSLHESTKYRHFNKIR    60
PfCD01_010017400-t41_1-p1      MKNIKNMKNIKSEGFIFFVLVFFICIIFGCIYESLHEGPYKKTLNSLHESTKYRHFNKIR    60
PfSD01_010016600-t41_1-p1      MKNIKNMKNIKSEGFIFFVLVFFICIIFGCIYESLHEGPYKKTLNSLHESTKYRHFNKIR    60
PfKH01_010018100-t41_1-p1      MKNIKNMKNIKSEGFIFFVLVFFICIIFGCIYESLHEGPYKKTLNSLHESTKYRHFNKIR    60
PfGA01_010017200-t41_1-p1      MKNIKNMKNIKSEGFIFFVLVFFICIIFGCIYESLHEGPYKKTLNSLHESTKYRHFNKIR    60
PfGN01_010017400-t41_1-p1      MKNIKNMKNIKSEGFIFFVLVFFICIIFGCIYESLHEGPYKKTLNSLHESTKYRHFNKIR    60
PfDd2_010016800-t41_1-p1       MKNIKNMKNIKSEGFIFFVLVFFICIIFGCIYESLHEGPYKKTLNSLHESTKYRHFNKIR    60
PfML01_010016900-t41_1-p1      MKNIKNMKNIKSEGFIFFVLVFFICIIFGCIYESLHEGPYKKTLNSLHESTKYRHFNKIR    60
PRCDC_0111600.1-p1             ------MKNIKSEGFIFFVLVFFICIIFGCIYESLHEYPYKKALNCLHESEKFREFNKIR    54
PRG01_0113700-t36_1-p1         ------MKNIKSEGFIFFVLVFFICIIFGCIYESLHEYPYKKALNCLHESEKFREFNKIR    54
                                     ***** * ::**:::**: ** ***:*.::*  *:*  :  * * *:*.*****

PADL01_0110900-t36_1-p1        LLSENKNILQFNKEKISLHDYINNDNYNTVDANDYNCYKDKEEQCNDSSC-VDKKNYNRS    113
PGABG01_0111700-t36_1-p1       LLSENKNILEFNKEKISLHDYINNDNYNTLDAIDYNFYNDKEEQCNDTTC-VDKKNYNRT    113
PGSY75_0113300-t31_1-p1        LLSENKNILEFNKEKISLHDYINNDNYNTLDAIDYNFYNDKEEQCNDTTC-VDKKNYNRT    113
SPJ08214.1                     LLTEYKNTLQLKEEKLSLHDYINNDRYNNIDTNDHTLCKDTEEQFNDTTS-VDKKNEKVT    113
PF3D7_0113300.1-p1             LLTEYKDTLQIKVEQKSLRDYVNNDRYNNVNTNDYTSYKDKGEQFNDTICVVDKKKENVT    120
PfNF54_010017900.1-p1          LLTEYKDTLQIKVEQKSLRDYVNNDRYNNVNTNDYTSYKDKGEQFNDTICVVDKKKENVT    120
PfGB4_010017100-t41_1-p1       LLTEYKDTLQIKVEQKSLRDYVNNDRYNNVNTNDYTSYKDKGEQFNDTICVVDKKKENVT    120
SOS76152.1                     LLTEYKDTLQIKVEQKSLRDYVNNDRYNNVNTNDYTSYNDKGEQFNDTICVVDKKKENVT    120
PfHB3_010017200-t41_1-p1       LLTEYKDTLQIKVEQKSLRDYVNNDRYNNVNTNDYTSYKDKGEQFNDTICVVDKKKENVT    120
PfTG01_010018100-t41_1-p1      LLTEYKDTLQIKVEQKSLRDYVNNDRYNNVNTNDYTSYKDKGEQFNDTICVVDKKKENVT    120
PfNF135_010017200.1-p1         LLTEYKDTLQIKVEQKSLRDYVNNDRYNNVNTNDYTSYKDKGEQFNDTICVVDKKKENVT    120
PfKH02_010016900-t41_1-p1      LLTEYKDTLQIKVEQKSLRDYVNNDRYNNVNTNDYTSYKDKGEQFNDTICVVDKKKENVT    120
PfNF166_010016800.1-p1         LLTEYKDTLQIKVEQKSLRDYVNNDRYNNVNTNDYTSYKDKGEQFNDTICVVDKKKENVT    120
Pf7G8-2_000046900.1-p1         LLTEYKDTLQIKVEQKSLRDYVNNDRYNNVNTNDYTSYKDKGEQFNDTICVVDKKKENVT    120
Pf7G8_010017300-t41_1-p1       LLTEYKDTLQIKVEQKSLRDYVNNDRYNNVNTNDYTSYKDKGEQFNDTICVVDKKKENVT    120
PfKE01_010016300-t41_1-p1      LLTEYKDTLQIKVEQKSLRDYVNNDRYNNVNTNDYTSYKDKGEQFNDTICVVDKKKENVT    120
PfIT_010016400-t41_1-p1        LLTEYKDTLQIKVEQKSLRDYVNNDRYNNVNTNDYTSYKDKGEQFNDTICVVDKKKENVT    120
PfSN01_010015800-t41_1-p1      LLTEYKDTLQIKVEQKSLRDYVNNDRYNNVNTNDYTSYKDKGEQFNDTICVVDKKKENVT    120
PfCD01_010017400-t41_1-p1      LLTEYKDTLQIKVEQKSLRDYVNNDRYNNVNTNDYTSYKDKGEQFNDTICVVDKKKENVT    120
PfSD01_010016600-t41_1-p1      LLTEYKDTLQIKVEQKSLRDYVNNDRYNNVNTNDYTSYKDKGEQFNDTICVVDKKKENVT    120
PfKH01_010018100-t41_1-p1      LLTEYKDTLQIKVEQKSLRDYVNNDRYNNVNTNDYTSYKDKGEQFNDTICVVDKKKENVT    120
PfGA01_010017200-t41_1-p1      LLTEYKDTLQIKVEQKSLRDYVNNDRYNNVNTNDYTSYKDKGEQFNDTICVVDKKKENVT    120
PfGN01_010017400-t41_1-p1      LLTEYKDTLQIKVEQKSLRDYVNNDRYNNVNTNDYTSYKDKGEQFNDTICVVDKKKENVT    120
PfDd2_010016800-t41_1-p1       LLTEYKDTLQIKVEQKSLRDYVNNDRYNNVNTNDYTSYKDKGEQFNDTICVVDKKKENVT    120
PfML01_010016900-t41_1-p1      LLTEYKDTLQIKVEQKSLRDYVNNDRYNNVNTNDYTSYKDKGEQFNDTICVVDKKKENVT    120
PRCDC_0111600.1-p1             LLTEYKDTLQIKEEKFTLHDYVNNDRYNNVNTNNYTLYKDKEEQFNDTICVVDKKKENGT    114
PRG01_0113700-t36_1-p1         LLTEYKDTLQIKEEKFTLHDYVNNDRYNNVNTNNYTLYKDKEEQFNDTICVVDKKKENGT    114
                               **:* *: *::: *: :*:**:***.**.::: ::.  :*. ** **: . ****: : :
```

```
PADL01_0110900-t36_1-p1        NNNEEECSKNFYQYLKYMEQNVNVNTNNKNINDKNINDNNTELQIISNDHNTKNYKYDDN    173
PGABG01_0111700-t36_1-p1       NNNEEECSKNFYQYLKYMEQNVNVNTNN----------INTELQIISNDHNKQNYKYDNN    163
PGSY75_0113300-t31_1-p1        NNNEEECSKNFYQYLKYMEQNVNVNTNN----------INTELQIISNDHNKQNYNYDNN    163
SPJ08214.1                     TNNDEKCNKNFYQYLQYLEHNVNGYTNNKNTEL----------QIVSND-----------    152
PF3D7_0113300.1-p1             INNEEECNKNFYQYLQYLEHNNK-------------------------------------    143
PfNF54_010017900.1-p1          INNEEECNKNFYQYLQYLEHNNK-------------------------------------    143
PfGB4_010017100-t41_1-p1       INNEEECNKNFYQYLQYLEHNNK-------------------------------------    143
SOS76152.1                     INNEEECNKNFYQYLQYLEHNNK-------------------------------------    143
PfHB3_010017200-t41_1-p1       INNEEECNKNFYQYLQYLEHNNK-------------------------------------    143
PfTG01_010018100-t41_1-p1      INNEEECNKNFYQYLQYLEHNNK-------------------------------------    143
PfNF135_010017200.1-p1         INNEEECNKNFYQYLQYLEHNNK-------------------------------------    143
PfKH02_010016900-t41_1-p1      INNEEECNKNFYQYLQYLEHNNK-------------------------------------    143
PfNF166_010016800.1-p1         INNEEECNKNFYQYLQYLEHNNK-------------------------------------    143
Pf7G8-2_000046900.1-p1         INNEEECNKNFYQYLQYLEHNNK-------------------------------------    143
Pf7G8_010017300-t41_1-p1       INNEEECNKNFYQYLQYLEHNNK-------------------------------------    143
PfKE01_010016300-t41_1-p1      INNEEECNKNFYQYLQYLEHNNK-------------------------------------    143
PfIT_010016400-t41_1-p1        INNEEECNKNFYQYLQYLEHNNK-------------------------------------    143
PfSN01_010015800-t41_1-p1      INNEEECNKNFYQYLQYLEHNNK-------------------------------------    143
PfCD01_010017400-t41_1-p1      INNEEECNKNFYQYLQYLEHNNK-------------------------------------    143
PfSD01_010016600-t41_1-p1      INNEEECNKNFYQYLQYLEHNNK-------------------------------------    143
PfKH01_010018100-t41_1-p1      INNEEECNKNFYQYLQYLEHNNK-------------------------------------    143
PfGA01_010017200-t41_1-p1      INNEEECNKNFYQYLQYLEHNNK-------------------------------------    143
PfGN01_010017400-t41_1-p1      INNEEECNKNFYQYLQYLEHNNK-------------------------------------    143
PfDd2_010016800-t41_1-p1       INNEEECNKNFYQYLQYLEHNNK-------------------------------------    143
PfML01_010016900-t41_1-p1      INNEEECNKNFYQYLQYLEHNNK-------------------------------------    143
PRCDC_0111600.1-p1             INNEEECNKNFYQYLQYLEHNVHGKENNNKYDN----------KYDNQNDNKYDSKY---    161
PRG01_0113700-t36_1-p1         INNEEECNKNFYQYLQYLEHNVHGKENNNKYDN----------KYDNQNDNKYDSKYDNQ    164
                                **:*:*.*******:*:*:* :

PADL01_0110900-t36_1-p1        NN-------------------------------SNNNNSEYGKTNNFLERNNKVIDSQQN    202
PGABG01_0111700-t36_1-p1       SDN-----------------------NN-NSDNNNNNNSEYGETNYFLERNNKLIDSQQN    199
PGSY75_0113300-t31_1-p1        SDN-----------------------NNNNSDNNNNNNSEYGETNYFLERNNKLIDSQQN    200
SPJ08214.1                     -----------------------QNIQYY----ENENKNENDQTNYFLGGNNKHMDSQQN    185
PF3D7_0113300.1-p1             ------------------------------------QDNKYEETNYFLQGNDKHIDSEHN    167
PfNF54_010017900.1-p1          ------------------------------------QDNKYEETNYFLQGNDKHIDSEHN    167
PfGB4_010017100-t41_1-p1       ------------------------------------QDNKYEETNYFLQGNDKHIDSEHN    167
SOS76152.1                     ------------------------------------QDNKYEETNYFLQGNDKHIDSEHN    167
PfHB3_010017200-t41_1-p1       ------------------------------------QDNKYEETNYFLQGNDKHIDSEHN    167
PfTG01_010018100-t41_1-p1      ------------------------------------QDNKYEETNYFLQGNDKHIDSEHN    167
PfNF135_010017200.1-p1         ------------------------------------QDNKYEETNYFLQGNDKHIDSEHN    167
PfKH02_010016900-t41_1-p1      ------------------------------------QDNKYEETNYFLQGNDKHIDSEHN    167
PfNF166_010016800.1-p1         ------------------------------------QDNKYEETNYFLQGNDKHIDSEHN    167
Pf7G8-2_000046900.1-p1         ------------------------------------QDNKYEETNYFLQGNDKHIDSEHN    167
Pf7G8_010017300-t41_1-p1       ------------------------------------QDNKYEETNYFLQGNDKHIDSEHN    167
PfKE01_010016300-t41_1-p1      ------------------------------------QDNKYEETNYFLQGNDKHIDSEHN    167
PfIT_010016400-t41_1-p1        ------------------------------------QDNKYEETNYFLQGNDKHIDSEHN    167
PfSN01_010015800-t41_1-p1      ------------------------------------QDNKYEETNYFLQGNDKHIDSEHN    167
PfCD01_010017400-t41_1-p1      ------------------------------------QDNKYEETNYFLQGNDKHIDSEHN    167
PfSD01_010016600-t41_1-p1      ------------------------------------QDNKYEETNYFLQGNDKHIDSEHN    167
PfKH01_010018100-t41_1-p1      ------------------------------------QDNKYEETNYFLQGNDKHIDSEHN    167
PfGA01_010017200-t41_1-p1      ------------------------------------QDNKYEETNYFLQGNDKHIDSEHN    167
PfGN01_010017400-t41_1-p1      ------------------------------------QDNKYEETNYFLQGNDKHIDSEHN    167
PfDd2_010016800-t41_1-p1       ------------------------------------QDNKYEETNYFLQGNDKHIDSEHN    167
PfML01_010016900-t41_1-p1      ------------------------------------QDNKYEETNYFLQGNDKHIDSEHN    167
PRCDC_0111600.1-p1             -DNQNDNKYDSKYDNQYDNKYDNQNDNKYDNQNDNQYDNKYDETNYFLWGNDKHIDSEHN    220
PRG01_0113700-t36_1-p1         YDNQNDNKYDSKYDNQYDNKYDNQNDNKYDNQNDNQYDNKYDETNYFLWGNDKHIDSEHN    224
                                                                    ..:  :** **  *:* :**::*
```

```
PADL01_0110900-t36_1-p1        KMNNIFKETINKTLTYDMSTDNSHTNNNSRHYENQNGNREYMNLSNDNLTYGNSPYDILP    262
PGABG01_0111700-t36_1-p1       EMNNIFQETINKTLTYDMSTENSYTNNNSMHYETQNGNREYMNLSNNNLTYIISPYDILP    259
PGSY75_0113300-t31_1-p1        EMNNIFQETINKTLTYDMSTENSHTNNNSIHYETQNGNREYMNLSNNNLTYIISPYDILP    260
SPJ08214.1                     GINKIYKETIHNTLTYNESTENTHTHNNLRDDKPQNGKMEYNNQSNNNLPYDNSPYNISP    245
PF3D7_0113300.1-p1             GINKMYKETIHKTLTSDVSTENSYTHNNSRDDEPQNGKRTYNNQSNNNLPYDNSSYNISP    227
PfNF54_010017900.1-p1          GINKMYKETIHKTLTSDVSTENSYTHNNSRDDEPQNGKRTYNNQSNNNLPYDNSSYNISP    227
PfGB4_010017100-t41_1-p1       GINKMYKETIHKTLTSDVSTENSYTHNNSRDDEPQNGKRTYNNQSNNNLPYDNSSYNISP    227
SOS76152.1                     GINKMYKETIHKTLTSDVSTENSYTHNNSRDDEPQNGKRTYNNQSNNNLPYDNSSYNISP    227
PfHB3_010017200-t41_1-p1       GINKMYKETIHKTLTSDVSTENSYTHNNSRDDEPQNGKRTYNNQSNNNLPYDNSSYNISP    227
PfTG01_010018100-t41_1-p1      GINKMYKETIHKTLTSDVSTENSYTHNNSRDDEPQNGKRTYNNQSNNNLPYDNSSYNISP    227
PfNF135_010017200.1-p1         GINKMYKETIHKTLTSDVSTENSYTHNNSRDDEPQNGKRTYNNQSNNNLPYDNSSYNISP    227
PfKH02_010016900-t41_1-p1      GINKMYKETIHKTLTSDVSTENSYTHNNSRDDEPQNGKRTYNNQSNNNLPYDNSSYNISP    227
PfNF166_010016800.1-p1         GINKMYKETIHKTLTSDVSTENSYTHNNSRDDEPQNGKRTYNNQSNNNLPYDNSSYNISP    227
Pf7G8-2_000046900.1-p1         GINKMYKETIHKTLTSDVSTENSYTHNNSRDDEPQNGKRTYNNQSNNNLPYDNSSYNISP    227
Pf7G8_010017300-t41_1-p1       GINKMYKETIHKTLTSDVSTENSYTHNNSRDDEPQNGKRTYNNQSNNNLPYDNSSYNISP    227
PfKE01_010016300-t41_1-p1      GINKMYKETIHKTLTSDVSTENSYTHNNSRDDEPQNGKRTYNNQSNNNLPYDNSSYNISP    227
PfIT_010016400-t41_1-p1        GINKMYKETIHKTLTSDVSTENSYTHNNSRDDEPQNGKRTYNNQSNNNLPYDNSSYNISP    227
PfSN01_010015800-t41_1-p1      GINKMYKETIHKTLTSDVSTENSYTHNNSRDDEPQNGKRTYNNQSNNNLPYDNSSYNISP    227
PfCD01_010017400-t41_1-p1      GINKMYKETIHKTLTSDVSTENSYTHNNSRDDEPQNGKRTYNNQSNNNLPYDNSSYNISP    227
PfSD01_010016600-t41_1-p1      GINKMYKETIHKTLTSDVSTENSYTHNNSRDDEPQNGKRTYNNQSNNNLPYDNSSYNISP    227
PfKH01_010018100-t41_1-p1      GINKMYKETIHKTLTSDVSTENSYTHNNSRDDEPQNGKRTYNNQSNNNLPYDNSSYNISP    227
PfGA01_010017200-t41_1-p1      GINKMYKETIHKTLTSDVSTENSYTHNNSRDDEPQNGKRTYNNQSNNNLPYDNSSYNISP    227
PfGN01_010017400-t41_1-p1      GINKMYKETIHKTLTSDVSTENSYTHNNSRDDEPQNGKRTYNNQSNNNLPYDNSSYNISP    227
PfDd2_010016800-t41_1-p1       GINKMYKETIHKTLTSDVSTENSYTHNNSRDDEPQNGKRTYNNQSNNNLPYDNSSYNISP    227
PfML01_010016900-t41_1-p1      GINKMYKETIHKTLTSDVSTENSYTHNNSRDDEPQNGKRTYNNQSNNNLPYDNSSYNISP    227
PRCDC_0111600.1-p1             GINKIYKETIHKALTSDVSTENSNTHNNSRDGEPQNGKLTYNNQSNNNLTYDNSSYNITP    280
PRG01_0113700-t36_1-p1         GINKIYKETIHKALTSDVSTENSNTHNNSRDGEPQNGKLTYNNQSNNNLTYDNSSYNITP    284
                                :*::::***:::** : **:*: *:**  . : ***:  * * **:** *  * *:* *

PADL01_0110900-t36_1-p1        YN---------DQNNLSQYETVDSNHCNDLNIYNPYYESIDLPYHESTDLPYHEPTDLPY    313
PGABG01_0111700-t36_1-p1       YN---------DQNNLSEYETLDSNHCNDLNIYNPYYQPI--------------------    290
PGSY75_0113300-t31_1-p1        YN---------DQNNLSQYETLDSNHCNDLNIYNPYYQPI--------------------    291
SPJ08214.1                     YNGSNNHVPYNTSNNFSQYNNPDNKYCYYLNIYHPYYGSMNYPHHRLEHYSH---H----    298
PF3D7_0113300.1-p1             YHGPNNNVPYNKSNNFEQCNTQDNKHCNDLDTYHTCYGPDNYPQKYDNYRQECDNYRQEC    287
PfNF54_010017900.1-p1          YHGPNNNVPYNKSNNFEQCNTQDNKHCNDLDTYHTCYGPDNYPQKYDNYRQECDNYRQEC    287
PfGB4_010017100-t41_1-p1       YHGPNNNVPYNKSNNFEQCNTQDNKHCNDLDTYHTCYGPDNYPQKYDNYRQEC-------    280
SOS76152.1                     YHGPNNNVPYNKSNNFEQCNTQDNKHCNDLDTYHTCYGPDNYPQKYDNYRQEYDNYRQEC    287
PfHB3_010017200-t41_1-p1       YHGPNNNVPYNKSNNFEQCNTQDNKHCNDLDTYHTCYGPDNYPQKYDNY-------RQEC    280
PfTG01_010018100-t41_1-p1      YHGPNNNVPYNKSNNFEQCNTQDNKHCNDLDTYHTCYGPDNYPQKYDNY-------RQEC    280
PfNF135_010017200.1-p1         YHGPNNNVPYNKSNNFEQCNTQDNKHCNDLDTYYTCYGPDNYPQKYDNY-------RQEC    280
PfKH02_010016900-t41_1-p1      YHGPNNNVPYNKSNNFEQCNTQDNKHCNDLDTYHTCYGPDNYPQKYDNY-------RQEC    280
PfNF166_010016800.1-p1         YHGPNNNVPYNKSNNFEQCNTQDNKHCNDLDTYHTCYGPDNYPQKYDNY-------RQEC    280
Pf7G8-2_000046900.1-p1         YHGPNNNVPYNKSNNFEQCNTQDNKHCNDLDTYHTCYGPDNYPQKYDNY-------RQEC    280
Pf7G8_010017300-t41_1-p1       YHGPNNNVPYNKSNNFEQCNTQDNKHCNDLDTYHTCYGPDNYPQKYDNY-------RQEC    280
PfKE01_010016300-t41_1-p1      YHGPNNNVPYNKSNNFEQCNTQDNKHCNDLDTYHTCYGPDNYPQKYDNY-------RQEC    280
PfIT_010016400-t41_1-p1        YHGPNNNVPYNKSNNFEQCNTQDNKHCNDLDTYHTCYGPDNYPQKYDNY-------RQEC    280
PfSN01_010015800-t41_1-p1      YHGPNNNVPYNKSNNFEQCNTQDNKHCNDLDTYHTCYGPDNYPQKYDNY-------RQEC    280
PfCD01_010017400-t41_1-p1      YHGPNNNVPYNKSNNFEQCNTQDNKHCNDLDTYHTCYGPDNYPQKYDNY-------RQEC    280
PfSD01_010016600-t41_1-p1      YHGPNNNVPYNKSNNFEQCNTQDNKHCNDLDTYHTCYGPDNYPQKYDNY-------RQEC    280
PfKH01_010018100-t41_1-p1      YHGPNNNVPYNKSNNFEQCNTQDNKHCNDLDTYYTCYGPDNYPQKYDNY-------RQEC    280
PfGA01_010017200-t41_1-p1      YHGPNNNVPYNKSNNFEQCNTQDNKHCNDLDTYHTCYGPDNYPQKYDNY-------RQEC    280
PfGN01_010017400-t41_1-p1      YHGPNNNVPYNKSNNFEQCNTQDNKHCNDLDTYHTCYGPDNYPQKYDNY-------RQEC    280
PfDd2_010016800-t41_1-p1       YHGPNNNVPYNKSNNFEQCNTQDNKHCNDLDTYHTCYGPDNYPQKYDNY-------RQEC    280
PfML01_010016900-t41_1-p1      YHGPNNNVPYNKSNNFEQCNTQDNKHCNDLDTYHTCYGPDNYPQKYDNY-------RQEC    280
PRCDC_0111600.1-p1             YHGPNNNIPYNKSNNSEQCNTQDNKHCNALDIYHTSYGPDNYPHGYDNHTHGYDNHPHGY    340
PRG01_0113700-t36_1-p1         YHGPNNNIPYNKSNNSEQCNTQDNKHCNALDIYHTSYGPDNYPHGYDNHTHGYDNHPHGY    344
                               *:          .** .: :. *.::*  *: *   *
```

```
PADL01_0110900-t36_1-p1        H----EPTDLPYH---EPTDLP-------YY--EPVSYTHDRSNNYSNRNYSNNN-Y---    353
PGABG01_0111700-t36_1-p1       -------------------DLP-------YY--EPVSYAHDRSNNYSHHSNHNYSHH---    319
PGSY75_0113300-t31_1-p1        -------------------DLP-------YY--EPVSYAHDRSNNYSHHSNHNYSHH---    320
SPJ08214.1                     ---------RPDHYSH-------------------------------HRPDH--------    310
PF3D7_0113300.1-p1             DNYRQEYDNYPQKYDNYRQECDNYRQ----------------------------------    313
PfNF54_010017900.1-p1          DNYRQEYDNYPQKYDNYRQECDNYRQ----------------------------------    313
PfGB4_010017100-t41_1-p1       DNYRQEYDNYPQKYDNYRQECDNYRQ----------------------------------    306
SOS76152.1                     DNYPQEYDNYPQEYDNYPHGFDNYPRGFDNYPRGFDN-----------------------    324
PfHB3_010017200-t41_1-p1       DNYRQEYDNYPHGFDNYPRGFDNYPRGFDNYPHGFDN-----------------------    317
PfTG01_010018100-t41_1-p1      DNYRQECDNYRQEYDNYPHGFDNYPRGFDNYPHGFDN-----------------------    317
PfNF135_010017200.1-p1         DNYRQKYDNYPHGFDNYPRGFDNYPHGFDNYPRGF-------------------------    315
PfKH02_010016900-t41_1-p1      DNYRQECDNYPHGFDNYPRGFDNYPRGFDNYPRGF-------------------------    315
PfNF166_010016800.1-p1         DNYRQEYDNYPHGFDNYPRGFDNYPHGFDNYPRGF-------------------------    315
Pf7G8-2_000046900.1-p1         DNYRQEYDNYPHGFDNYPRGFDNYPRGFDNYPRGFDN-----------------------    317
Pf7G8_010017300-t41_1-p1       DNYRQEYDNYPHGFDNYPRGFDNYPRGFDNYPRGFDN-----------------------    317
PfKE01_010016300-t41_1-p1      DNYRQECDNYPHGFDNYPRGFDNYPRGFDNYPRGFDN-----------------------    317
PfIT_010016400-t41_1-p1        DNYRQEYDNYPHGFDNYPHGFDNYP-------RGFDN-----------------------    310
PfSN01_010015800-t41_1-p1      DNYRQEYDNYPHGFDNYPRGFDNYP-------RGFDN-----------------------    310
PfCD01_010017400-t41_1-p1      DNYRQEYDNYPHGFDNYPRGFDNYP-------RGFDN-----------------------    310
PfSD01_010016600-t41_1-p1      DNYRQEYDNYPHGFDNYPRGFDNYP-------RGFDN-----------------------    310
PfKH01_010018100-t41_1-p1      DNYRQEYDNYPHGFDNYPRGFDNYP-------R---------------------------    306
PfGA01_010017200-t41_1-p1      DNYRKECDNYPHGFDNYPRGFDNYP-------R---------------------------    306
PfGN01_010017400-t41_1-p1      DNYRQECDNYPHGFDNYPRGFDNYP-------R---------------------------    306
PfDd2_010016800-t41_1-p1       DNYRQEYDNYPHGFDNYPRGFDNYP-------H---------------------------    306
PfML01_010016900-t41_1-p1      DNYRQEYDNYPHGFDNYPRGFDNYP-------H---------------------------    306
PRCDC_0111600.1-p1             DNHTHGYDNHPHGYDNHPHGYDKHPHGPDNHP----------------------------    372
PRG01_0113700-t36_1-p1         DNHPHGYDNHPHGYDNHPHGYDKHPHGYDNYPHRPHIYPHG-YDNYPHRPHIYPHGYDNH    403

PADL01_0110900-t36_1-p1        --SNRNY------SNNNYSKHNYPHH-------------NYSHHNYPHHNYPHHNYPHHN    392
PGABG01_0111700-t36_1-p1       --SNHNYHHHSNHNHSHHSNHNYPHH-------------SNHNHNHSHHNHSH-----HN    359
PGSY75_0113300-t31_1-p1        --SNHNYSHHSNHNYSHHSNHNYPHH-------------SNHNHNHSHHNHPHHNHSHHN    365
SPJ08214.1                     ------YSH--------HKPDHYLH------HKPDHYL------HHKPDHY------LHH    338
PF3D7_0113300.1-p1             ------------------EYDNYPHGFDNYPRGFDNYPHGYDNHPHRPHIYPHGFDNHPH    355
PfNF54_010017900.1-p1          ------------------EYDNYPHGFDNYPRGFDNYPHGYDNHPHRPHIYPHGFDNHPH    355
PfGB4_010017100-t41_1-p1       ------------------EYDNYPHGFDNYPRGFDNYPHGYDNHPHRPHIYPHGFDNHPH    348
SOS76152.1                     ------YPRGF--DNYPHGFDNYPHGFDNYPHGFDNYPHGYDNHPHRPHIYPHGFDNHPH    376
PfHB3_010017200-t41_1-p1       ------YPHGF--DNYPHGFDNYPHGYDNHPHRPHIYPHGFDNHPHRPHIYPHGFDNHPH    369
PfTG01_010018100-t41_1-p1      ------YPHGF--DNYPHGFDNYPHGYDNHPHRPHIY--------------PHGFDNHPH    355
PfNF135_010017200.1-p1         -------------DNYPRGFDNYPHGYDNHPHRPHIY--------------PHGFDNHPH    348
PfKH02_010016900-t41_1-p1      -------------DNYPRGFDNYPHGYDNHPHRPHIY--------------PHGFDNHPH    348
PfNF166_010016800.1-p1         -------------DNYPRGFDNYPHGYDNHPHRPHIY--------------PHGFDNHPH    348
Pf7G8-2_000046900.1-p1         ------YPHGF--DNYPHGFDNYPHGYDNHPHRPHIY--------------PHGFDNHPH    355
Pf7G8_010017300-t41_1-p1       ------YPHGF--DNYPHGFDNYPHGYDNHPHRPHIY--------------PHGFDNHPH    355
PfKE01_010016300-t41_1-p1      ------YPHGF--DNYPHGFDNYPHGYDNHPHRPHIY--------------PHGFDNHPH    355
PfIT_010016400-t41_1-p1        ------YPHGF--DNYPHGFDNYPHGYDNHPHRPHIY--------------PHGFDNHPH    348
PfSN01_010015800-t41_1-p1      ------YPHGF--DNYPHGFDNYPHGYDNHPHRPHIY--------------PHGFDNHPH    348
PfCD01_010017400-t41_1-p1      ------YPHGF--DNYPHGFDNYPHGYDNHPHRPHIY--------------PHGFDNHPH    348
PfSD01_010016600-t41_1-p1      ------YPHGF--DNYPHGFDNYPHGYDNHPHRPHIY--------------PHGFDNHPH    348
PfKH01_010018100-t41_1-p1      ---------GF--DNYPRGFDNYPHGYDNHPHRPHIY--------------PHGFDNHPH    341
PfGA01_010017200-t41_1-p1      ---------GF--DNYPRGFDNYPHGYDNHPHRPHIY--------------PHGFDNHPH    341
PfGN01_010017400-t41_1-p1      ---------GF--DNYPHGFDNYPHGYDNHPHRPHIY--------------PHGFDNHPH    341
PfDd2_010016800-t41_1-p1       ---------GF--DNYPHGFDNYPHGYDNHPHRPHIY--------------PHGFDNHPH    341
PfML01_010016900-t41_1-p1      ---------GF--DNYPHGFDNYPHGYDNHPHRPHIY--------------PHGFDNHPH    341
PRCDC_0111600.1-p1             --------HGY--DNHPHGYDNHPHGYDNYPHRPHIYPHGYDNHPHRPHIYPHGYDNHPH    422
PRG01_0113700-t36_1-p1         PHRPHIYPHGY--DNHPHRPHIYPHGYDNHPHRPHIYPHGYDNHPHRPHIYPHGYDNHPH    461
                                                   . : *                                  :
```

```
PADL01_0110900-t36_1-p1        YPHH---NYPHHRYGSSGDPYHRHDHIMDRSYYYSNLQNDNHDMMLTYIPKNDNKSLYDE    449
PGABG01_0111700-t36_1-p1       HSHHNHSHRSHHRYGPGGDPYHRHDHIMDRSYYNNNIQNDNDDMMLTYNQMNDNKSLYDE    419
PGSY75_0113300-t31_1-p1        HSHHNHSHHNHHRYGPGGDPYHRHDHIMDRSYYNNNLQNDNDDMMLTYNQMNDNKSLYDE    425
SPJ08214.1                     RQ-NKYSHNLSLRNISVGGPYYRPAHMMERFDYYSNLKNDAHYMMLPYNRMNDNKSMCDE    397
PF3D7_0113300.1-p1             RP-HMYPHNFPMRNESVGGPYYRPPHIIERSNYYKNPKKAPHNMMLPCDTMKDNKSICDE    414
PfNF54_010017900.1-p1          RP-HMYPHNFPMRNESVGGPYYRPPHIIERSNYYKNPKKAPHNMMLPCDTMKDNKSICDE    414
PfGB4_010017100-t41_1-p1       RP-HMYPHNFPMRNESVGGPYYRPPHIIERSNYYRNPKKAPHNMMLPCDTMKDNKSICDE    407
SOS76152.1                     RP-HMYPHNFPMRNESVGGPYYRPPHIIERSNYYRNPKKAPHNMMLPCDTMKDNKSICDE    435
PfHB3_010017200-t41_1-p1       RP-HMYPHNFPMRNESVGGPYYRPPHIIERSNYYRNPKKAPHNMMLPCDTMKDNKSICDE    428
PfTG01_010018100-t41_1-p1      RP-HMYPHNFPMRNESVGGPYYRPPHIIERSNYYRNPKKAPHNMMLPCDTMKDNKSICDE    414
PfNF135_010017200.1-p1         RP-HMYPHNFPMRNESVGGPYYRPPHIIERSNYYRNPKKAPHNMMLPCDTMKDNKSICDE    407
PfKH02_010016900-t41_1-p1      RP-HMYPHNFPMRNESVGGPYYRPPHIIERSNYYRNPKKAPHNMMLPCDTMKDNKSICDE    407
PfNF166_010016800.1-p1         RP-HMYPHNFPMRNESVGGPYYRPPHIIERSNYYRNPKKAPHNMMLPCDTMKDNKSICDE    407
Pf7G8-2_000046900.1-p1         RP-HMYPHNFPMRNESVGGPYYRPPHIIERSNYYKNPKKAPHNMMLPCDTMKDNKSICDE    414
Pf7G8_010017300-t41_1-p1       RP-HMYPHNFPMRNESVGGPYYRPPHIIERSNYYKNPKKAPHNMMLPCDTMKDNKSICDE    414
PfKE01_010016300-t41_1-p1      RP-HMYPHNFPMRNESVGGPYYRPPHIIERSNYYRNPKKAPHNMMLPCDTMKDNKSICDE    414
PfIT_010016400-t41_1-p1        RP-HMYPHNFPMRNESVGGPYYRPPHIIERSNYYRNPKKAPHNMMLPCDTMKDNKSICDE    407
PfSN01_010015800-t41_1-p1      RP-HMYPHNFPMRNESVGGPYYRPPHIIERSNYYRNPKKAPHNMMLPCDTMKDNKSICDE    407
PfCD01_010017400-t41_1-p1      RP-HMYPHNFPMRNESVGGPYYRPPHIIERSNYYRNPKKAPHNMMLPCDTMKDNKSICDE    407
PfSD01_010016600-t41_1-p1      RP-HMYPHNFPMRNESVGGPYYRPPHIIERSNYYRNPKKAPHNMMLPCDTMKDNKSICDE    407
PfKH01_010018100-t41_1-p1      RP-HMYPHNFPMRNESVGGPYYRPPHIIERSNYYRNPKKAPHNMMLPCDTMKDNKSICDE    400
PfGA01_010017200-t41_1-p1      RP-HMYPHNFPMRNESVGGPYYRPPHIIERSNYYRNPKKAPHNMMLPCDTMKDNKSICDE    400
PfGN01_010017400-t41_1-p1      RP-HMYPHNFPMRNESVGGPYYRPPHIIERSNYYRNPKKAPHNMMLPCDTMKDNKSICDE    400
PfDd2_010016800-t41_1-p1       RP-HMYPHNFPMRNESVGGPYYRPPHIIERSNYYRNPKKAPHNMMLPCDTMKDNKSICDE    400
PfML01_010016900-t41_1-p1      RP-HMYPHNFPMRNESVGGPYYRPPHIIERSNYYRNPKKAPHNMMLPCDTMKDNKSICDE    400
PRCDC_0111600.1-p1             RP-HIYPHNFPMRNESVGGPYYRPPHLIERSDYYSNPKNEPHNMMLPYNTMEDNTSICDE    481
PRG01_0113700-t36_1-p1         RP-HIYPHNFPMRNESVGGPYYRPPHLIERSDYYSNPKNEPHNMMLPYNTMEDNTSICDE    520
                                  :   :    *    *.**:*  *:::*  *  * ::  . ***     :**.*: **

PADL01_0110900-t36_1-p1        QNFELELKKIIKKNHLQNDNITDSYDTAVNDFNKKLKEYNKKLNEYNEKLNEYTS-RLNE    508
PGABG01_0111700-t36_1-p1       QNFELELKKIIKKNHLQNSNITDSCDTRVSDYNKKLVEYNKKLNEYNEKLNEYTR-RLNE    478
PGSY75_0113300-t31_1-p1        QNFELELKKIIKKNHLQNGNITDSCDTRVSDYNKKLVEYNKKLNEYNEKLNEYTR-RLNE    484
SPJ08214.1                     QNFQLGLEKMKKKNNLQNGNIRDSHDTRISDYNKRLNGYNKRLNGYNKKNSYNKQSSYNK    457
PF3D7_0113300.1-p1             QNFQRELEKIIKKNNLQNGNIRDNHDTRINDYNKRLTEYNKRLTEYNKRLTEYTK-RLNE    473
PfNF54_010017900.1-p1          QNFQRELEKIIKKNNLQNGNIRDNHDTRINDYNKRLTEYNKRLTEYNKRLTEYTK-RLNE    473
PfGB4_010017100-t41_1-p1       QNFQRELEKIIKKNNLQNGNIRDNHDTRINDYNKRLTEYNKRLTEYNKRLTEYTK-RLNE    466
SOS76152.1                     QNFQRELEKIIKKNNLQNGNSRDNHDTRINDYNKRLTEYNKRLTEYNKRLTEYTK-RLNE    494
PfHB3_010017200-t41_1-p1       QNFQRELEKIIKKNNLQNGNIRDNHDTRINDYNKRLTEYNKRLTAYNKRLTEYTK-RLNE    487
PfTG01_010018100-t41_1-p1      QNFQRELEKIIKKNNLQNGNIRDNHDTRINDYNKRLTEYNKRLTEYNKRLTEYTK-RLNE    473
PfNF135_010017200.1-p1         QNFQRELEKIIKKNNLQNGNIRDNHDTRINDYNKRLTEYNKRLTEYNKRLTEYTK-RLNE    466
PfKH02_010016900-t41_1-p1      QNFQRELEKIIKKNNLQNGNIRDNHDTRINDYNKRLTEYNKRLTEYNKRLTEYTK-RLNE    466
PfNF166_010016800.1-p1         QNFQRELEKIIKKNNLQNGNIRDNHDTRINDYNKRLTEYNKRLTEYNKRLTEYTK-RLNE    466
Pf7G8-2_000046900.1-p1         QNFQRELEKIIKKNNLQNGNIRDNHDTRINDYNKRLTEYNKRLTEYNKRLTEYTK-RLNE    473
Pf7G8_010017300-t41_1-p1       QNFQRELEKIIKKNNLQNGNIRDNHDTRINDYNKRLTEYNKRLTEYNKRLTEYTK-RLNE    473
PfKE01_010016300-t41_1-p1      QNFQRELEKIIKKNNLQNGNIRDNHDTRINDYNKRLTEYNKRLTEYNKRLTEYTK-RLNE    473
PfIT_010016400-t41_1-p1        QNFQRELEKIIKKNNLQNGNIRDNHDTRINDYNKRLTEYNKRLTEYNKRLTEYTK-RLNE    466
PfSN01_010015800-t41_1-p1      QNFQRELEKIIKKNNLQNGNIRDNHDTRINDYNKRLTEYNKRLTEYNKRLTEYTK-RLNE    466
PfCD01_010017400-t41_1-p1      QNFQRELEKIIKKNNLQNGNIRDNHDTRINDYNKRLTEYNKRLTEYNKRLTEYTK-RLNE    466
PfSD01_010016600-t41_1-p1      QNFQRELEKIIKKNNLQNGNIRDNHDTRINDYNKRLTEYNKRLTEYNKRLTEYTK-RLNE    466
PfKH01_010018100-t41_1-p1      QNFQRELEKIIKKNNLQNGNIRDNHDTRINDYNKRLTEYNKRLTEYNKRLTEYTK-RLNE    459
PfGA01_010017200-t41_1-p1      QNFQRELEKIIKKNNLQNGNIRDNHDTRINDYNKRLTEYNKRLTEYNKRLTEYTK-RLNE    459
PfGN01_010017400-t41_1-p1      QNFQRELEKIIKKNNLQNGNIRDNHDTRINDYNKRLTEYNKRLTEYNKRLTEYTK-RLNE    459
PfDd2_010016800-t41_1-p1       QNFQRELEKIIKKNNLQNGNIRDNHDTRINDYNKRLTEYNKRLTEYNKRLTEYTK-RLNE    459
PfML01_010016900-t41_1-p1      QNFQRELEKIIKKNNLQNGNIRDNHDTRINDYNKRLTEYNKRLTEYNKRLTEYTK-RLNE    459
PRCDC_0111600.1-p1             QSFQLELEKIIKKNNLQNGNIRDSHDTGINDYDKRLTEYNKRLTEYNKRLNEYTK-RLNE    540
PRG01_0113700-t36_1-p1         QSFQLELEKIIKKNNLQNGNIRDSHDTGINDYDKRLTEYNKRLTEYNKRLNEYTK-RLNE    579
                               *.*:  *:*: ***:***.*  *. ** :.*::*:*  ***:*. **:: .  .    *:
```

```
PADL01_0110900-t36_1-p1        YNKKHNEKKKNDDNKKSGQNNNENILSQDIVLYGTDFQNAFRYRQNTRSYYSNIYSNREE    568
PGABG01_0111700-t36_1-p1       YNKKHNEN------KQSRQNNNENTLSQDIVLYGADFQNAFRYRQNTRSYYSNIHSNREE    532
PGSY75_0113300-t31_1-p1        YNKKHNEN------KQSRQNNNENTLSQDIVLYGADFQNAFRYRQNTRSYYSNIHSNREE    538
SPJ08214.1                     QSS------YNKQNCYNKQNGIENRSSNNIVLYGNNFQNAFRYRQNTRSYYTNIYSNGEA    511
PF3D7_0113300.1-p1             HYK------RNGYNIQNRQNSIERAQSNDVVLYGHNFQNAFRYKQNTRSYYPHVNSNEAT    527
PfNF54_010017900.1-p1          HYK------RNGYNIQNRQNSIERAQSNDVVLYGHNFQNAFRYKQNTRSYYPHVNSNEAT    527
PfGB4_010017100-t41_1-p1       HYK------RKGYNIQNRQNSIERAQSNDVVLYGHNFQNAFRYKQNTRSYYPHVNSNEAT    520
SOS76152.1                     HYK------RNGYNIQNRQNSIERAPSNDVVLYGHNFQNAFRYKQNTRSYYPHVNSNEAT    548
PfHB3_010017200-t41_1-p1       HYK------RNGYNIQNRQNSIERAQSNDVVLYGHNFQNAFRYKQNTRSYYPHVNSNEAT    541
PfTG01_010018100-t41_1-p1      HYK------RKGYNIQNRQNSIERAQSNDVVLYGHNFQNAFRYKQNTRSYYPHVNSNEAT    527
PfNF135_010017200.1-p1         HYK------RNGYNIQNRQNSIERAQSNDVVLYGHNFQNAFRYKQNTRSYYPHVNSNEAT    520
PfKH02_010016900-t41_1-p1      HYK------RNGYNIQNRQNSIERAQSNDVVLYGHNFQNAFRYKQNTRSYYPHVNSNEAT    520
PfNF166_010016800.1-p1         HYK------RKGYNIQNRQNSIERAQSNDVVLYGHNFQNAFRYKQNTRSYYPHVNSNEAT    520
Pf7G8-2_000046900.1-p1         HYK------RKGYNIQNRQNSIERAQSNDVVLYGHNFQNAFRYKQNTRSYYPHVNSNEAT    527
Pf7G8_010017300-t41_1-p1       HYK------RKGYNIQNRQNSIERAQSNDVVLYGHNFQNAFRYKQNTRSYYPHVNSNEAT    527
PfKE01_010016300-t41_1-p1      HYK------RKGYNIQNRQNSIERAQSNDVVLYGHNFQNAFRYKQNTRSYYPHVNSNEAT    527
PfIT_010016400-t41_1-p1        HYK------RNGYNIQNRQNSIERAQSNDVVLYGHNFQNAFRYKQNTRSYYPHVNSNEAT    520
PfSN01_010015800-t41_1-p1      HYK------RNGYNIQNRQNSIERAQSKDVVLYGHNFQNAFRYKQNTRSYYPHVNSNEAT    520
PfCD01_010017400-t41_1-p1      HYK------RNGYNIQNRQNSIERAQSKDVVLYGHNFQNAFRYKQNTRSYYPHVNSNEAT    520
PfSD01_010016600-t41_1-p1      HYK------RNGYNIQNRQNSIERAQSNDVVLYGHNFQNAFRYKQNTRSYYPHVNSNEAT    520
PfKH01_010018100-t41_1-p1      HYK------RNGYNIQNRQNSIERAQSNDVVLYGHNFQNAFRYKQNTRSYYPHVNSNEAT    513
PfGA01_010017200-t41_1-p1      HYK------RKGYNIQNRQNSIERAQSNDVVLYGHNFQNAFRYKQNTRSYYPHVNSNEAT    513
PfGN01_010017400-t41_1-p1      HYK------RNGYNIQNRQNSIERAQSNDVVLYGHNFQNAFRYKQNTRSYYPHVNSNEAT    513
PfDd2_010016800-t41_1-p1       HYK------RNGYNIQNRQNSIERAQSNDVVLYGHNFQNAFRYKQNTRSYYPHVNSNEAT    513
PfML01_010016900-t41_1-p1      HYK------RKGYNIQNRQNSIERAQSNDVVLYGHNFQNAFRYKQNTRSYYPHVNSNEAT    513
PRCDC_0111600.1-p1             HYK------RSGYNIQNRQNSIEHIPSNDIVLYGHNFQNAFRYKQNTRSYYPHVNSKGTA    594
PRG01_0113700-t36_1-p1         HYK------RSGYNIQNRQNSIEHIPSNDIVLYGHNFQNAFRYKQNTRSYYPHVNSKGTA    633
                                 .             . **.* .  *:::**** :*******:******* :: *:

PADL01_0110900-t36_1-p1        DLDKVIDFTHNNNSSEDEYTFRNKQEIYHQHSKRLEKKLFDYQNGSNPVINFLERHF       625
PGABG01_0111700-t36_1-p1       DLDRVIDFTNNNNNSSEDEYTFGNEQEIYHQHSKRLEKKLFDYQNGTNPLINFLERHF      589
PGSY75_0113300-t31_1-p1        DLDRVIDFTNNNNNASEDEYTFGNEQEIYHQHSKRLEKKLFDYQNGTNPLINFLERHF      595
SPJ08214.1                     NHERARYFNQQNNRPQEEYPIKSEQHMHHVQSKRLDKKLYDYENCSNPVRNFLKRHF       568
PF3D7_0113300.1-p1             HHQKTMYFTQQNNYSREEYPIKSEQHLYHVKSKRLEKKLYDYQNGTNPVTNFLERHF       584
PfNF54_010017900.1-p1          HHQKTMYFTQQNNYSREEYPIKSEQHLYHVKSKRLEKKLYDYQNGTNPVTNFLERHF       584
PfGB4_010017100-t41_1-p1       HHQKTMYFTQQNNYSREEYPIKSEQHLYHVKSKRLEKKLYDYQNGTNPVTNFLERHF       577
SOS76152.1                     HHQKTMYFTQQNNYSREEYPIKSEQHLYHVKSKRLEKKLYDYQNGTNPVTNFLERHF       605
PfHB3_010017200-t41_1-p1       HHQKTMYFTQQNNYSREEYPIKSEQHLYHVKSKRLEKKLYDYQNGTNPVTNFLERHF       598
PfTG01_010018100-t41_1-p1      HHQKTMYFTQQNNYSREEYPIKSEQHLYHVKSKRLEKKLYDYQNGTNPVTNFLERHF       584
PfNF135_010017200.1-p1         HHQKTMYFTQQNNYSREEYPIKSEQHLYHVKSKRLEKKLYDYQNGTNPVTNFLERHF       577
PfKH02_010016900-t41_1-p1      HHQKTMYFTQQNNYSREEYPIKSEQHLYHVKSKRLEKKLYDYQNGTNPVTNFLERHF       577
PfNF166_010016800.1-p1         HHQKTMYFTQQNNYSREEYPIKSEQHLYHVKSKRLEKKLYDYQNGTNPVTNFLERHF       577
Pf7G8-2_000046900.1-p1         HHQKTMYFTQQNNYSREEYPIKSEQHLYHVKSKRLEKKLYDYQNGTNPVTNFLERHF       584
Pf7G8_010017300-t41_1-p1       HHQKTMYFTQQNNYSREEYPIKSEQHLYHVKSKRLEKKLYDYQNGTNPVTNFLERHF       584
PfKE01_010016300-t41_1-p1      HHQKTMYFTQQNNYSREEYPIKSEQHLYHVKSKRLEKKLYDYQNGTNPVTNFLERHF       584
PfIT_010016400-t41_1-p1        HHQKTMYFTQQNNYSREEYPIKSEQHLYHVKSKRLEKKLYDYQNGTNPVTNFLERHF       577
PfSN01_010015800-t41_1-p1      HHQKTMYFTQQNNYSREEYPIKSEQHLYHVKSKRLEKKLYDYQNGTNPVTNFLERHF       577
PfCD01_010017400-t41_1-p1      HHQKTMYFTQQNNYSREEYPIKSEQHLYHVKSKRLEKKLYDYQNGTNPVTNFLERHF       577
PfSD01_010016600-t41_1-p1      HHQKTMYFTQQNNYSREEYPIKSEQHLYHVKSKRLEKKLYDYQNGTNPVTNFLERHF       577
PfKH01_010018100-t41_1-p1      HHQKTMYFTQQNNYSREEYPIKSEQHLYHVKSKRLEKKLYDYQNGTNPVTNFLERHF       570
PfGA01_010017200-t41_1-p1      HHQKTMYFTQQNNYSREEYPIKSEQHLYHVKSKRLEKKLYDYQNGTNPVTNFLERHF       570
PfGN01_010017400-t41_1-p1      HHQKTMYFTQQNNYSREEYPIKSEQHLYHVKSKRLEKKLYDYQNGTNPVTNFLERHF       570
PfDd2_010016800-t41_1-p1       HHQKTMYFTQQNNYSREEYPIKSEQHLYHVKSKRLEKKLYDYQNGTNPVTNFLERHF       570
PfML01_010016900-t41_1-p1      HHQKTMYFTQQNNYSREEYPIKSEQHLYHVKSKRLEKKLYDYQNGTNPVTNFLERHF       570
PRCDC_0111600.1-p1             QHQKSMYFTQQNNYSQ-EYPTKSEQHMYHVKSKRLEKKLYDYQNGTNPITNFLERHF       650
PRG01_0113700-t36_1-p1         QHQKSMYFTQQNNYSQ-EYPTKSEQHMYHVKSKRLEKKLYDYQNGTNPITNFLERHF       689
                               . ::   *.::**  . **   .:*.::* :****:***:**:* :**: ***:***
```
