# Supplementary figures and images for: A Systematic Targeted Genetic Screen Identifies Proteins Involved in Cytoadherence of the Malaria Parasite P. falciparum
Source: Mol Microbiol. 2025 Jan 20;123(4):330–43. doi: 10.1111/mmi.15337 (PMC11976115; doi:10.1111/mmi.15337)

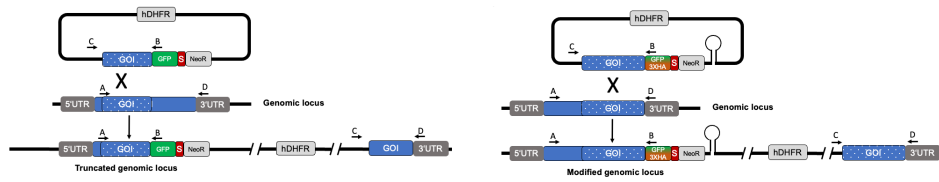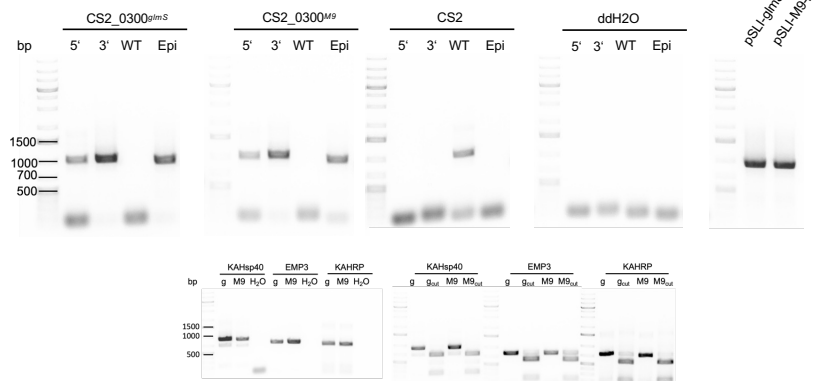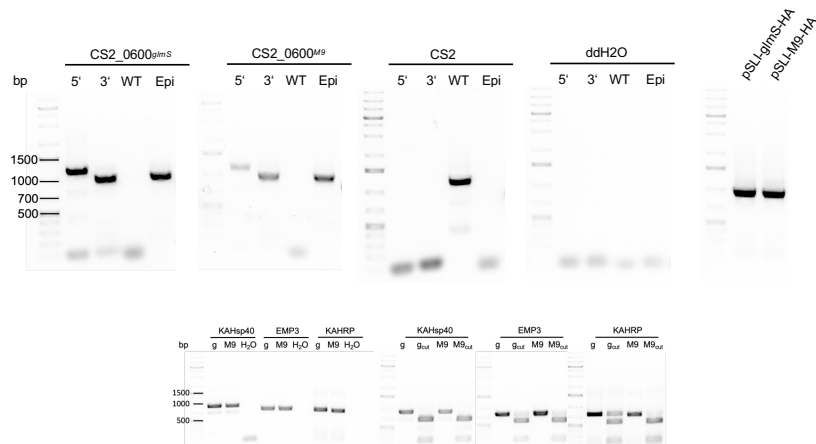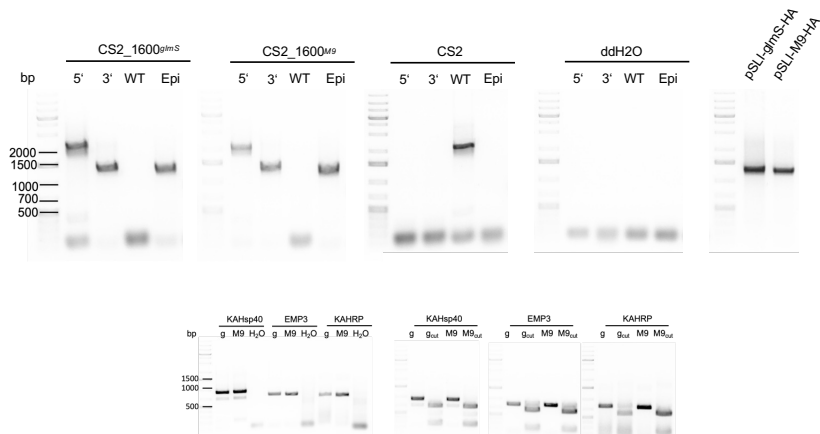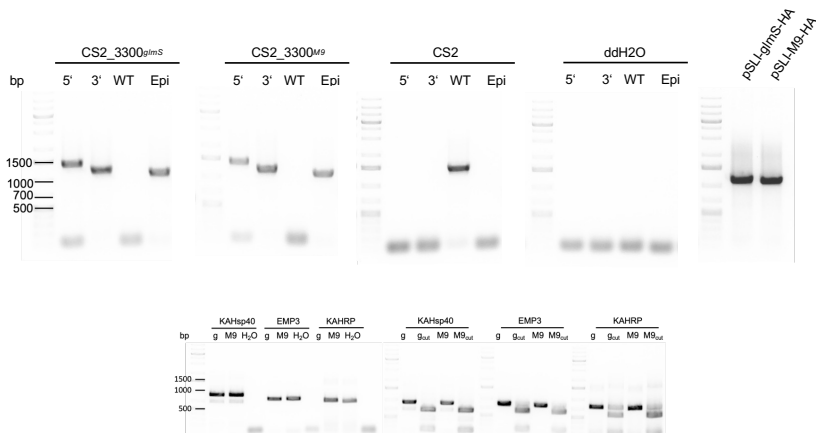

Supplement: Supplementary file 1 — Data S1 Generation of glmS knockdown cell lines. (Top) Strategy to generate glmS knockdown cell lines via SLI. The native genomic locus was modified to add an additional C‐terminal skip‐peptide (S) and HA‐tag or GFP‐tag to the GOI using homologous recombination and selection‐linked integration (SLI). UTR, untranslated region. Primers used for integration PCRs are indicated by arrows. (Lower panels) Verification of M9‐ and glmS knockdown cell lines via integration‐PCR. GlmS and M9 cell lines were tested for 5′ (primer A and B) and 3′ integration (primer C and D), episomal plasmids (primer C and B) and wild‐type‐locus (primer A and D). CS2, WT‐positive control; ddH20, negative control. Additionally, the integrity of the subtelomeric regions of chromosome 2, which harbours kahsp40, emp3 and kahrp, was verified by PCR and restriction digest. S2. Overview of gene regions targeted and primer binding sites. S3. Primers and antibodies used in this study. S4. Sequence analysis. S5. Middle section of deconvoluted Z‐stack from Figure 1. S6. Deconvoluted Z‐stack movie from Figure 1. S7. Solubility analysis. Infected erythrocytes were lysed in water by repeated freeze‐thawing, separated into a supernatant (SN) and pellet (P) fraction by centrifugation and prepared for analysis by Western blot. A total of 1 × 10^7^ parasite equivalents were loaded per lane. Positive controls (left lanes) are total parasite extracts. Antibodies against PfAldolase were used as a control for the soluble fraction and PfEXP1 for membrane‐bound proteins. S8. Light microscopy of parasite‐infected erythrocytes. Cell morphology and cell cycle progression of glmS and M9 cell lines were monitored for 72 h by imaging Giemsa‐stained blood smears of parasites at the time points and with the GlcN concentrations indicated. Scale bar, 5 μm. All images are representative of at least 10 independent observations. S9. Localisation of other exported proteins in M9 cell lines. Immunofluorescent localisation of PfKAHRP, P [file MMI-123-330-s001.zip › S1_Integration PCR.pdf]

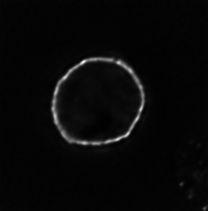

Supplement: Supplementary file 1 — Data S1 Generation of glmS knockdown cell lines. (Top) Strategy to generate glmS knockdown cell lines via SLI. The native genomic locus was modified to add an additional C‐terminal skip‐peptide (S) and HA‐tag or GFP‐tag to the GOI using homologous recombination and selection‐linked integration (SLI). UTR, untranslated region. Primers used for integration PCRs are indicated by arrows. (Lower panels) Verification of M9‐ and glmS knockdown cell lines via integration‐PCR. GlmS and M9 cell lines were tested for 5′ (primer A and B) and 3′ integration (primer C and D), episomal plasmids (primer C and B) and wild‐type‐locus (primer A and D). CS2, WT‐positive control; ddH20, negative control. Additionally, the integrity of the subtelomeric regions of chromosome 2, which harbours kahsp40, emp3 and kahrp, was verified by PCR and restriction digest. S2. Overview of gene regions targeted and primer binding sites. S3. Primers and antibodies used in this study. S4. Sequence analysis. S5. Middle section of deconvoluted Z‐stack from Figure 1. S6. Deconvoluted Z‐stack movie from Figure 1. S7. Solubility analysis. Infected erythrocytes were lysed in water by repeated freeze‐thawing, separated into a supernatant (SN) and pellet (P) fraction by centrifugation and prepared for analysis by Western blot. A total of 1 × 10^7^ parasite equivalents were loaded per lane. Positive controls (left lanes) are total parasite extracts. Antibodies against PfAldolase were used as a control for the soluble fraction and PfEXP1 for membrane‐bound proteins. S8. Light microscopy of parasite‐infected erythrocytes. Cell morphology and cell cycle progression of glmS and M9 cell lines were monitored for 72 h by imaging Giemsa‐stained blood smears of parasites at the time points and with the GlcN concentrations indicated. Scale bar, 5 μm. All images are representative of at least 10 independent observations. S9. Localisation of other exported proteins in M9 cell lines. Immunofluorescent localisation of PfKAHRP, P [file MMI-123-330-s001.zip › S5_Mid.tif]

**(a)**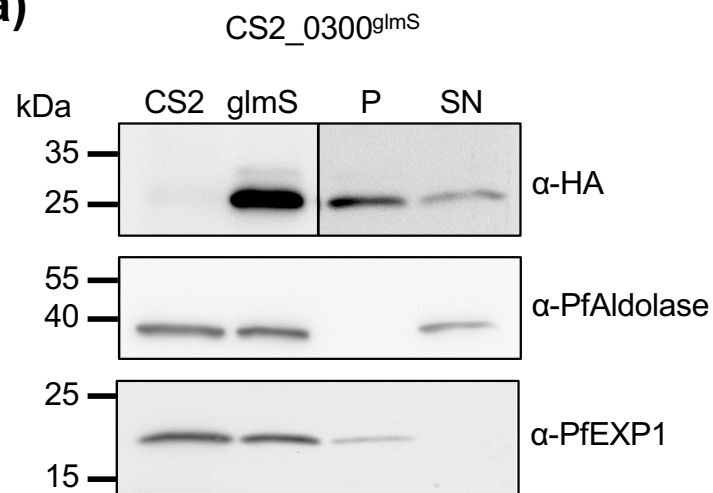**(b)**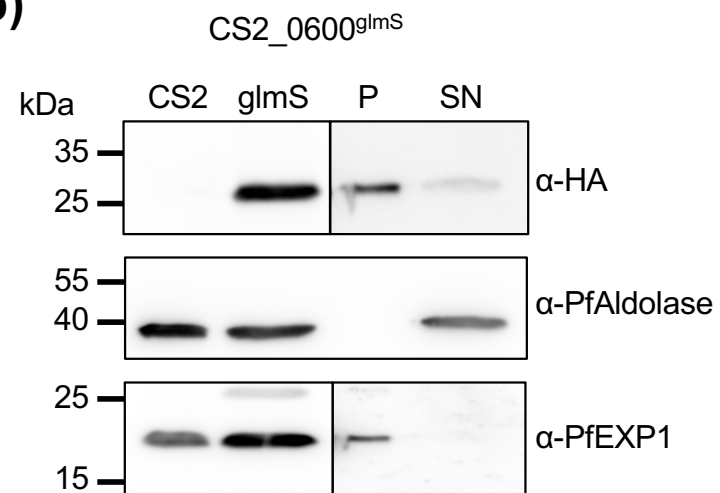**(c)**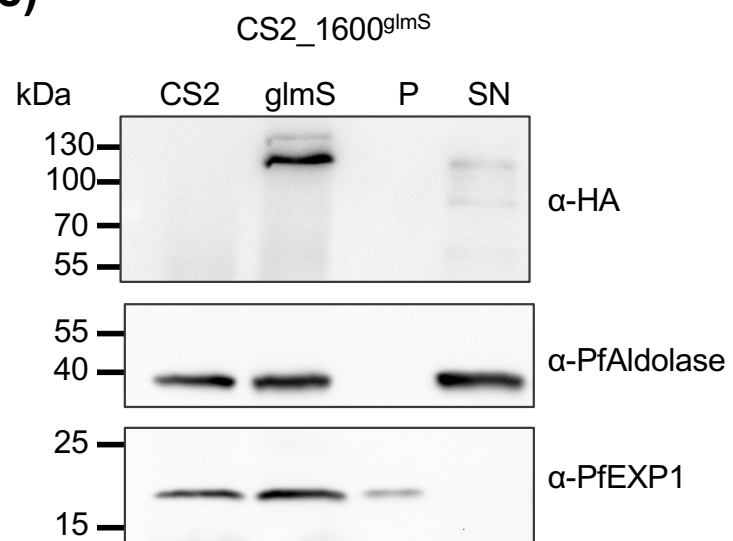**(d)**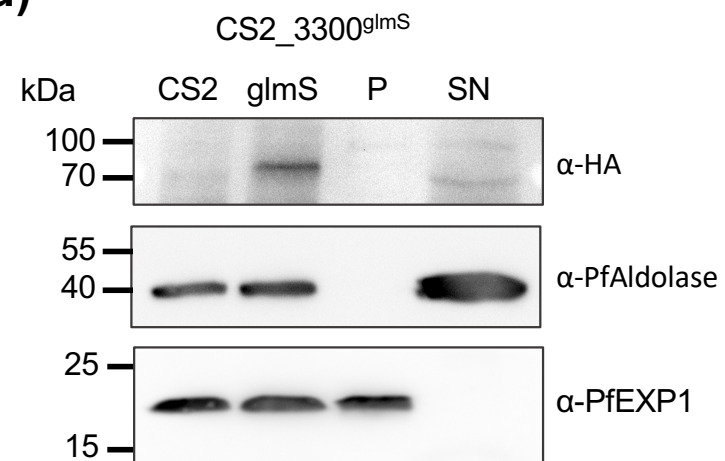

Supplement: Supplementary file 1 — Data S1 Generation of glmS knockdown cell lines. (Top) Strategy to generate glmS knockdown cell lines via SLI. The native genomic locus was modified to add an additional C‐terminal skip‐peptide (S) and HA‐tag or GFP‐tag to the GOI using homologous recombination and selection‐linked integration (SLI). UTR, untranslated region. Primers used for integration PCRs are indicated by arrows. (Lower panels) Verification of M9‐ and glmS knockdown cell lines via integration‐PCR. GlmS and M9 cell lines were tested for 5′ (primer A and B) and 3′ integration (primer C and D), episomal plasmids (primer C and B) and wild‐type‐locus (primer A and D). CS2, WT‐positive control; ddH20, negative control. Additionally, the integrity of the subtelomeric regions of chromosome 2, which harbours kahsp40, emp3 and kahrp, was verified by PCR and restriction digest. S2. Overview of gene regions targeted and primer binding sites. S3. Primers and antibodies used in this study. S4. Sequence analysis. S5. Middle section of deconvoluted Z‐stack from Figure 1. S6. Deconvoluted Z‐stack movie from Figure 1. S7. Solubility analysis. Infected erythrocytes were lysed in water by repeated freeze‐thawing, separated into a supernatant (SN) and pellet (P) fraction by centrifugation and prepared for analysis by Western blot. A total of 1 × 10^7^ parasite equivalents were loaded per lane. Positive controls (left lanes) are total parasite extracts. Antibodies against PfAldolase were used as a control for the soluble fraction and PfEXP1 for membrane‐bound proteins. S8. Light microscopy of parasite‐infected erythrocytes. Cell morphology and cell cycle progression of glmS and M9 cell lines were monitored for 72 h by imaging Giemsa‐stained blood smears of parasites at the time points and with the GlcN concentrations indicated. Scale bar, 5 μm. All images are representative of at least 10 independent observations. S9. Localisation of other exported proteins in M9 cell lines. Immunofluorescent localisation of PfKAHRP, P [file MMI-123-330-s001.zip › S7_solubility.pdf]

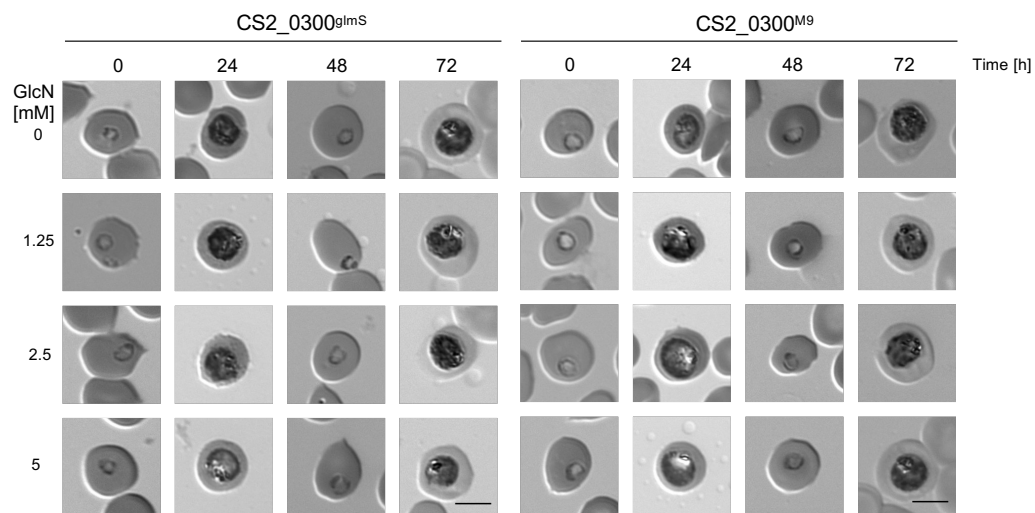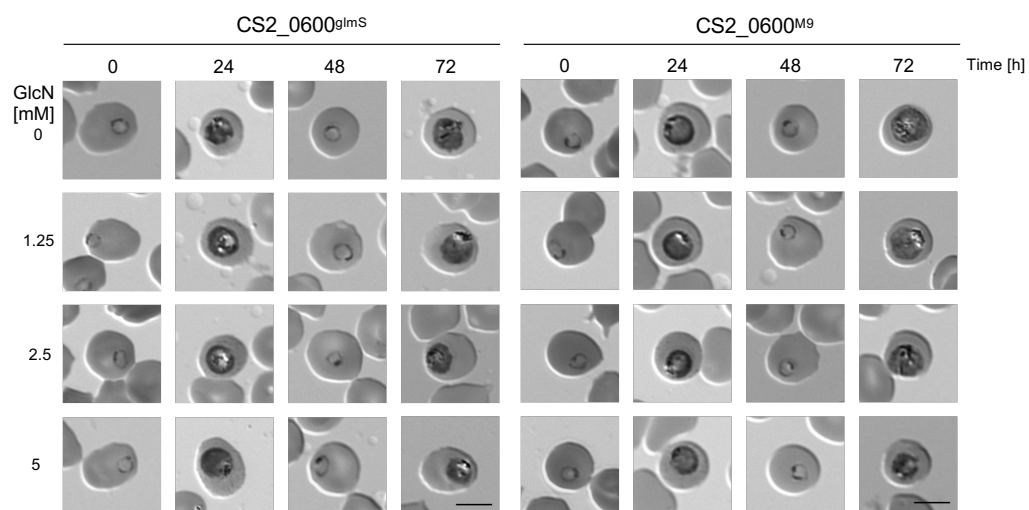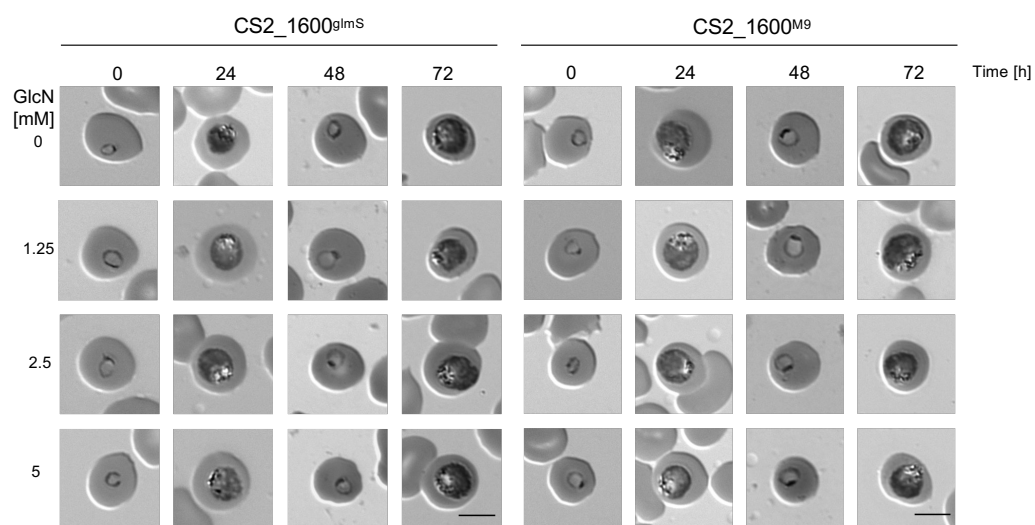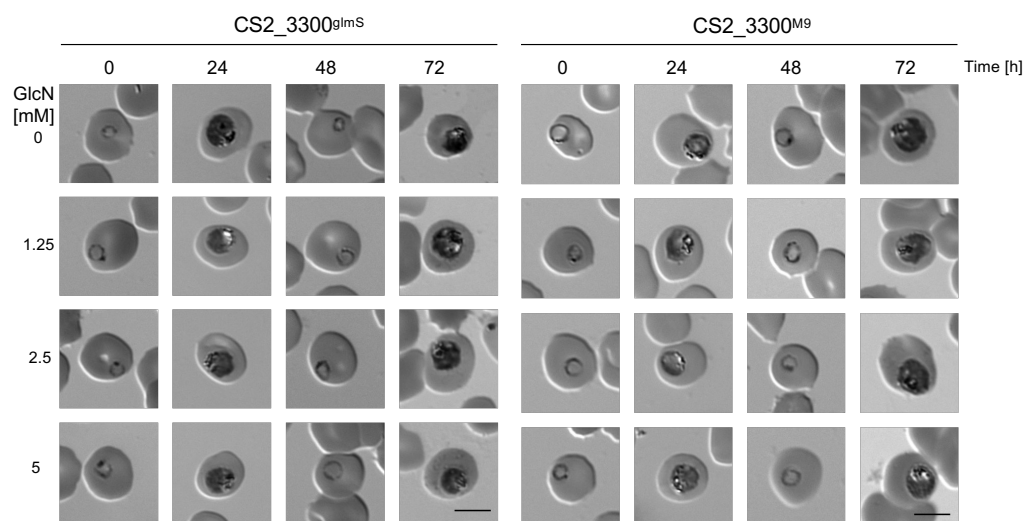

Supplement: Supplementary file 1 — Data S1 Generation of glmS knockdown cell lines. (Top) Strategy to generate glmS knockdown cell lines via SLI. The native genomic locus was modified to add an additional C‐terminal skip‐peptide (S) and HA‐tag or GFP‐tag to the GOI using homologous recombination and selection‐linked integration (SLI). UTR, untranslated region. Primers used for integration PCRs are indicated by arrows. (Lower panels) Verification of M9‐ and glmS knockdown cell lines via integration‐PCR. GlmS and M9 cell lines were tested for 5′ (primer A and B) and 3′ integration (primer C and D), episomal plasmids (primer C and B) and wild‐type‐locus (primer A and D). CS2, WT‐positive control; ddH20, negative control. Additionally, the integrity of the subtelomeric regions of chromosome 2, which harbours kahsp40, emp3 and kahrp, was verified by PCR and restriction digest. S2. Overview of gene regions targeted and primer binding sites. S3. Primers and antibodies used in this study. S4. Sequence analysis. S5. Middle section of deconvoluted Z‐stack from Figure 1. S6. Deconvoluted Z‐stack movie from Figure 1. S7. Solubility analysis. Infected erythrocytes were lysed in water by repeated freeze‐thawing, separated into a supernatant (SN) and pellet (P) fraction by centrifugation and prepared for analysis by Western blot. A total of 1 × 10^7^ parasite equivalents were loaded per lane. Positive controls (left lanes) are total parasite extracts. Antibodies against PfAldolase were used as a control for the soluble fraction and PfEXP1 for membrane‐bound proteins. S8. Light microscopy of parasite‐infected erythrocytes. Cell morphology and cell cycle progression of glmS and M9 cell lines were monitored for 72 h by imaging Giemsa‐stained blood smears of parasites at the time points and with the GlcN concentrations indicated. Scale bar, 5 μm. All images are representative of at least 10 independent observations. S9. Localisation of other exported proteins in M9 cell lines. Immunofluorescent localisation of PfKAHRP, P [file MMI-123-330-s001.zip › S8_DIC.pdf]

CS2\_0300<sup>M9</sup>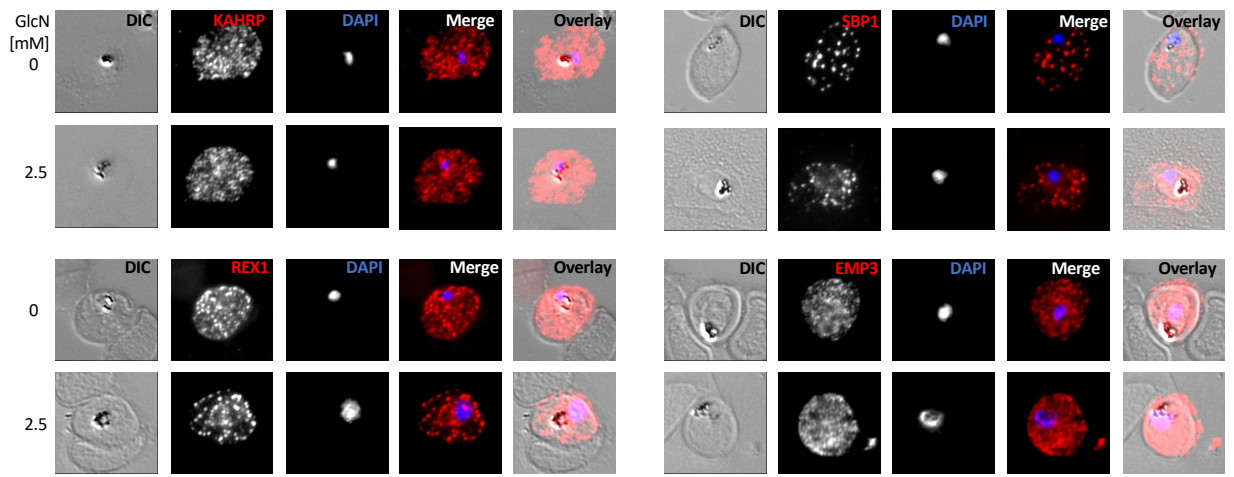CS2\_0600<sup>M9</sup>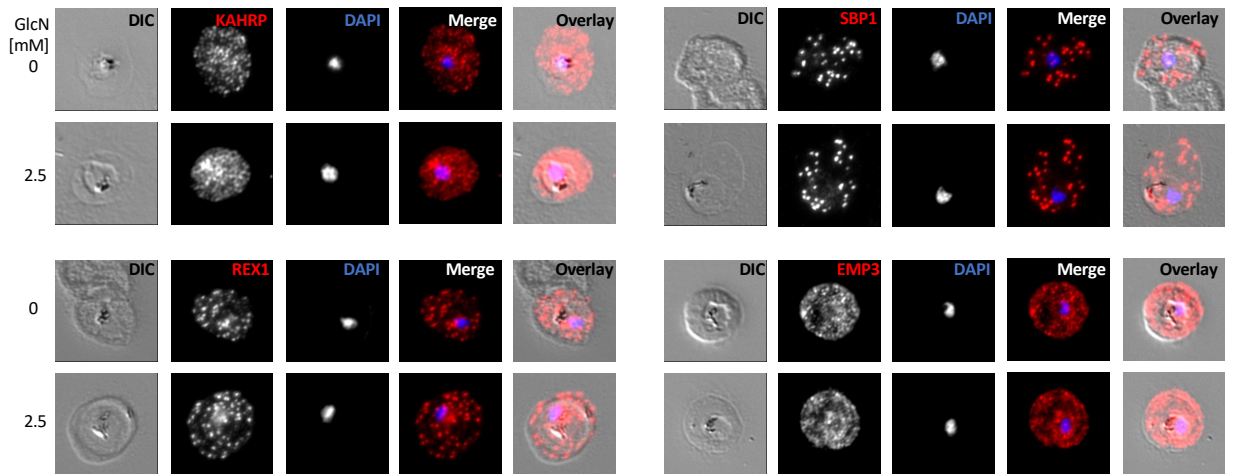CS2\_1600<sup>M9</sup>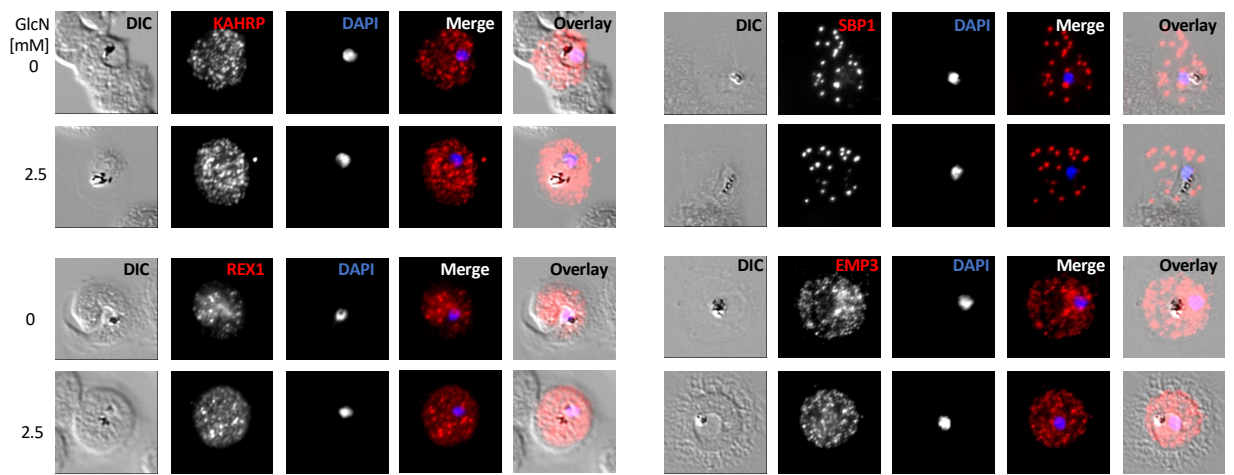CS2\_3300<sup>M9</sup>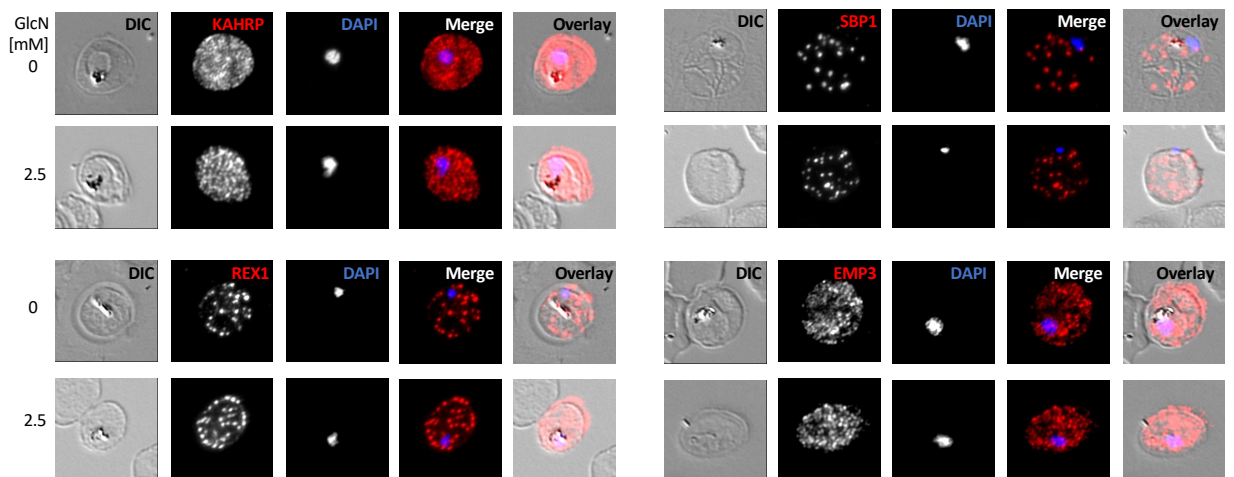

Supplement: Supplementary file 1 — Data S1 Generation of glmS knockdown cell lines. (Top) Strategy to generate glmS knockdown cell lines via SLI. The native genomic locus was modified to add an additional C‐terminal skip‐peptide (S) and HA‐tag or GFP‐tag to the GOI using homologous recombination and selection‐linked integration (SLI). UTR, untranslated region. Primers used for integration PCRs are indicated by arrows. (Lower panels) Verification of M9‐ and glmS knockdown cell lines via integration‐PCR. GlmS and M9 cell lines were tested for 5′ (primer A and B) and 3′ integration (primer C and D), episomal plasmids (primer C and B) and wild‐type‐locus (primer A and D). CS2, WT‐positive control; ddH20, negative control. Additionally, the integrity of the subtelomeric regions of chromosome 2, which harbours kahsp40, emp3 and kahrp, was verified by PCR and restriction digest. S2. Overview of gene regions targeted and primer binding sites. S3. Primers and antibodies used in this study. S4. Sequence analysis. S5. Middle section of deconvoluted Z‐stack from Figure 1. S6. Deconvoluted Z‐stack movie from Figure 1. S7. Solubility analysis. Infected erythrocytes were lysed in water by repeated freeze‐thawing, separated into a supernatant (SN) and pellet (P) fraction by centrifugation and prepared for analysis by Western blot. A total of 1 × 10^7^ parasite equivalents were loaded per lane. Positive controls (left lanes) are total parasite extracts. Antibodies against PfAldolase were used as a control for the soluble fraction and PfEXP1 for membrane‐bound proteins. S8. Light microscopy of parasite‐infected erythrocytes. Cell morphology and cell cycle progression of glmS and M9 cell lines were monitored for 72 h by imaging Giemsa‐stained blood smears of parasites at the time points and with the GlcN concentrations indicated. Scale bar, 5 μm. All images are representative of at least 10 independent observations. S9. Localisation of other exported proteins in M9 cell lines. Immunofluorescent localisation of PfKAHRP, P [file MMI-123-330-s001.zip › S9_M9_IFA.pdf]
